# Microbial community shifts elicit inflammation in the caecal mucosa via the GPR41/43 signalling pathway during subacute ruminal acidosis

**DOI:** 10.1186/s12917-019-2031-5

**Published:** 2019-08-19

**Authors:** Guangjun Chang, Huanmin Zhang, Yan Wang, Nana Ma, Roy Animesh Chandra, Gengping Ye, Su Zhuang, Weiyun Zhu, Xiangzhen Shen

**Affiliations:** 1 College of Veterinary Medicine, Nanjing Agricultural University, Nanjing, Jiangsu China; 2 Ranch Management Department, Bright Farming Co.,Ltd, Shanghai, China; 3 College of Animal Science and Technology, Nanjing Agricultural University, Nanjing, Jiangsu China

**Keywords:** Caecal microbiota, SCFAs, LPS, GPR41/GPR43, Signalling pathway, TLR4

## Abstract

**Background:**

Dietary structure in ruminants is closely connected with the composition of gastrointestinal microbiota. Merging study has shown that dietary induced SARA causes the alteration of microbial community in the cecum leading to the local inflammation. However, the mechanisms of cecum inflammation elicited by the shift of microbial flora in ruminants are largely unknown, and whether the development of this inflammation is modified by epigenetic modifications.

**Results:**

Ten multiparous lactating goats were randomly seperated into two groups and received either a low concentrate diet (LC, 40% concentrate, *n* = 5) or a high concentrate diet (HC, 60% concentrate) to induce subacute ruminal acidosis (SARA). Compared with LC, HC-induced SARA altered the predominant phyla and genera, thereby increasing the concentration of lipopolysaccharide (LPS) and short chain fatty acids (SCFAs). Meanwhile, HC-induced SARA enhanced the mRNA expression of cytokines and chemokines and the expression of mRNA and protein of GPR41, GPR43, p38 and ERK1/2, while HC-induced SARA had no effect on TLR4 and p65. Furthermore, HC-induced SARA decreased the percentage of chromatin compaction and DNA methylation at the area of the promoters of GPR41 and GPR43.

**Conclusion:**

This study indicated that HC diet induced SARA resulted in the alteration in the composition of cecal microbiota. This alteration increased the concentration of LPS, but failing to activate TLR4 signaling pathway due to the tolerance effect of intestinal epithelial cell to certain level of LPS, as well as elevated the concentration of SCFAs, thereby activating GPR41 and GPR43 signaling pathway to produce cytokines and chemokins and cause the cecal inflammation. And epigenetic mechanisms contributed to the development of this inflammation in the lactating goats suffering from SARA.

**Electronic supplementary material:**

The online version of this article (10.1186/s12917-019-2031-5) contains supplementary material, which is available to authorized users.

## Background

The population and composition of bacteria in the gastrointestinal tract (GT) of ruminants go together with the ingredients in the diet. High concentration (HC) diet feeding has become a popular practice to gain more economic benefits in the current intensive system of dairy production. However, emerging data has shown that the increased dietary concentrate, which raises the acidity and decreases bacterial richness and diversity in the GT of ruminants, disrupts the intestinal steady-state between the host and the microbiota [1]. Further studies confirm that long-term feeding of an HC diet induces subacute ruminal acidosis (SARA), which can result in an inflammatory response in the GT and uterus [2, 3], as well as the depression of milk quantity and quality and the increase of the culling rate in cows [4, 5], which ultimately causes large economic losses in the dairy industry. Although the effects of SARA on the GT barrier of ruminants have attracted considerable attention [6, 7], the detailed mechanism of the inflammatory injury in the GT barrier remains incompletely understood.

Previous studies documented the essentiality of the caecum as a major site of microbial fermentation to produce short-chain fatty acids (SCFAs) [8, 9]. Approximately 12% of the total SCFAs in ruminants originate from caecum microbial fermentation [10]. SCFAs, such as acetate, propionate and butyrate, have been verified to have wide impacts on various aspects of body physiology, which either positively or negatively regulate leukocyte degranulation and phagocytic functions and manipulate the proliferation, differentiation and apoptosis of epithelial cells [11, 12]. SCFAs also modulate diverse cellular functions (for example, synthesis and secretion) by binding to cell surface G-protein–coupled receptors (GPRs), such as GPR41 and GPR43. Recent evidence indicates that the activation of GPR41 and GPR43 by SCFAs elicits inflammatory responses in the GT [13, 14], but the regulatory mechanism of this inflammation is poorly understood.

Toll-like receptor 4, one of the pattern recognition molecules, is well-known as the specific receptor of lipopolysaccharide (LPS), and its activation orchestrates the expression of more than 100 immune-related genes in an NF-κB-dependent manner [15]. Previous studies revealed that HC diet-induced SARA causes a shift in the bacterial microbiota composition and increases SCFAs and LPS, leading to an inflammatory response in the caecum [6]. However, the pathogenesis of caecal inflammation elicited by SARA is unclear, and recent studies have shown that epithelial cells in the GT maintain immune tolerance to a certain level of LPS [16, 17]. Therefore, we hypothesized that the alterations of the microbial community elicit caecum mucosal inflammation via activating GPR41 and GRP43 signalling but may not elicit TLR4 signalling during SARA, and the expression of immune-related genes is modulated by epigenetic mechanisms. In the present study, lactating goats, the treated animal model, were fed a high concentrate diet (HC, 60% concentrate) to induce SARA or were fed a low concentrate diet (LC, 40% concentrate) as a control.

## Results

### Rumen pH value and milk parameters

**Fig. 1:**
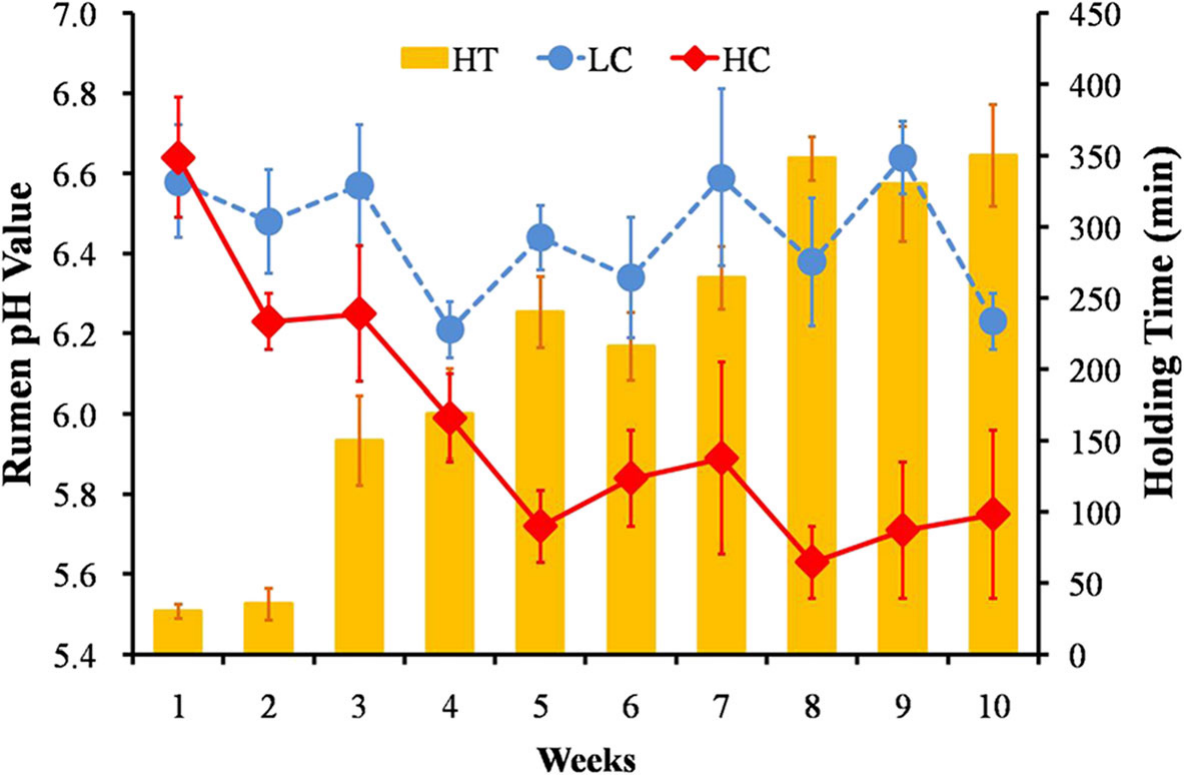
Rumen pH and duration time of pH < 5.6 of goats fed LC or HC diets. Blue circle indicates the variation tendency of rumen pH in the LC group (*n* = 5, mean ± SEM); red rhomboids indicates the variation tendency of rumen pH in the HC group (*n* = 5, mean ± SEM); yellow bar indicates the averaged duration time of rumen pH < 5.6 in the HC group. LC, low concentration diet; HC high concentration diet

Feeding an HC diet to lactating goats gradually reduced the averaged rumen pH value from 6.64 (1st week) to 5.75 (10th week). However, feeding an LC diet stably maintained the pH at approximately 6.2 throughout this experiment (Fig. 1). The duration that the average rumen pH value remained below 5.6 was more than 180 min/day in the HC group, from the 5^#^ week onward. The factors of diet and week significantly affected the rumen pH value (*P* < 0.01 and *P* = 0.03) in the HC group compared to that in LC group (Additional file 3: Table S3).

**Fig. 2:**
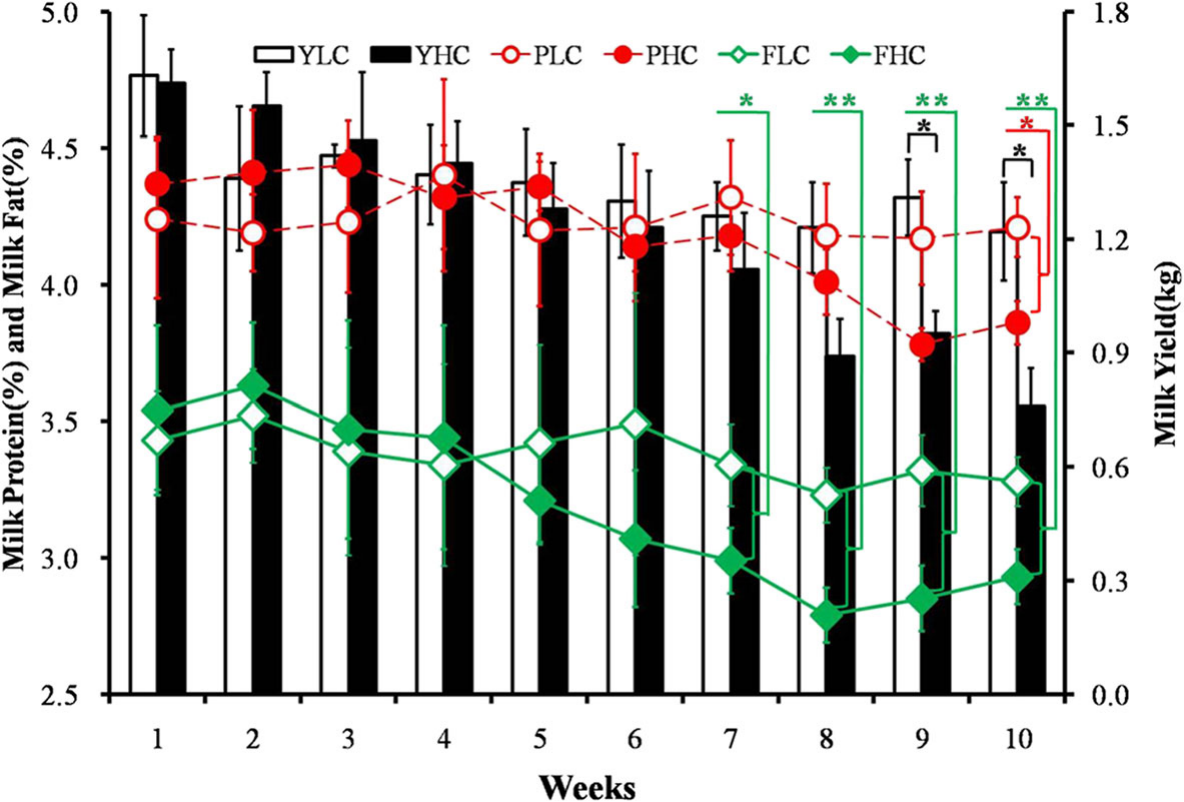
Milk yield and components of goats fed LC or HC diets for 10 weeks. YLC, PLC and FLC represent milk yield, milk protein and milk fat in the LC group (*n* = 5, mean ± SEM), respectively; YHC, PHC and FHC represent milk yield, milk protein and milk fat in the HC group (*n* = 5, mean ± SEM), respectively. Red circle and red filled circle indicate the variation tendency of milk protein; green rhomboids and green filled rhomboids indicate the variation tendency of milk fat; white and black filled bar indicate the weekly averaged milk yield of lactating goats from LC group or HC group. LC, low concentration diet; HC high concentration diet. Asterisks indicate the level of statistically significant difference: * *P* < 0.05; ** *P* < 0.01

Milk yield, milk protein, and milk fat displayed a gradual decreasing tendency in the HC group compared to those in the LC group. A significant difference in the average weekly milk yield between the LC group and the HC group was indicated at the 9^#^ and 10^#^ week, at the 10^#^ week for milk protein, and from the 7^#^ to the 10^#^ week for milk fat (Fig. 2). The diet significantly affected the milk yield, whereas milk protein and fat were not significantly affected across this experiment (Additional file 3: Table S3).

### Caecal microbiota community

**Fig. 3:**
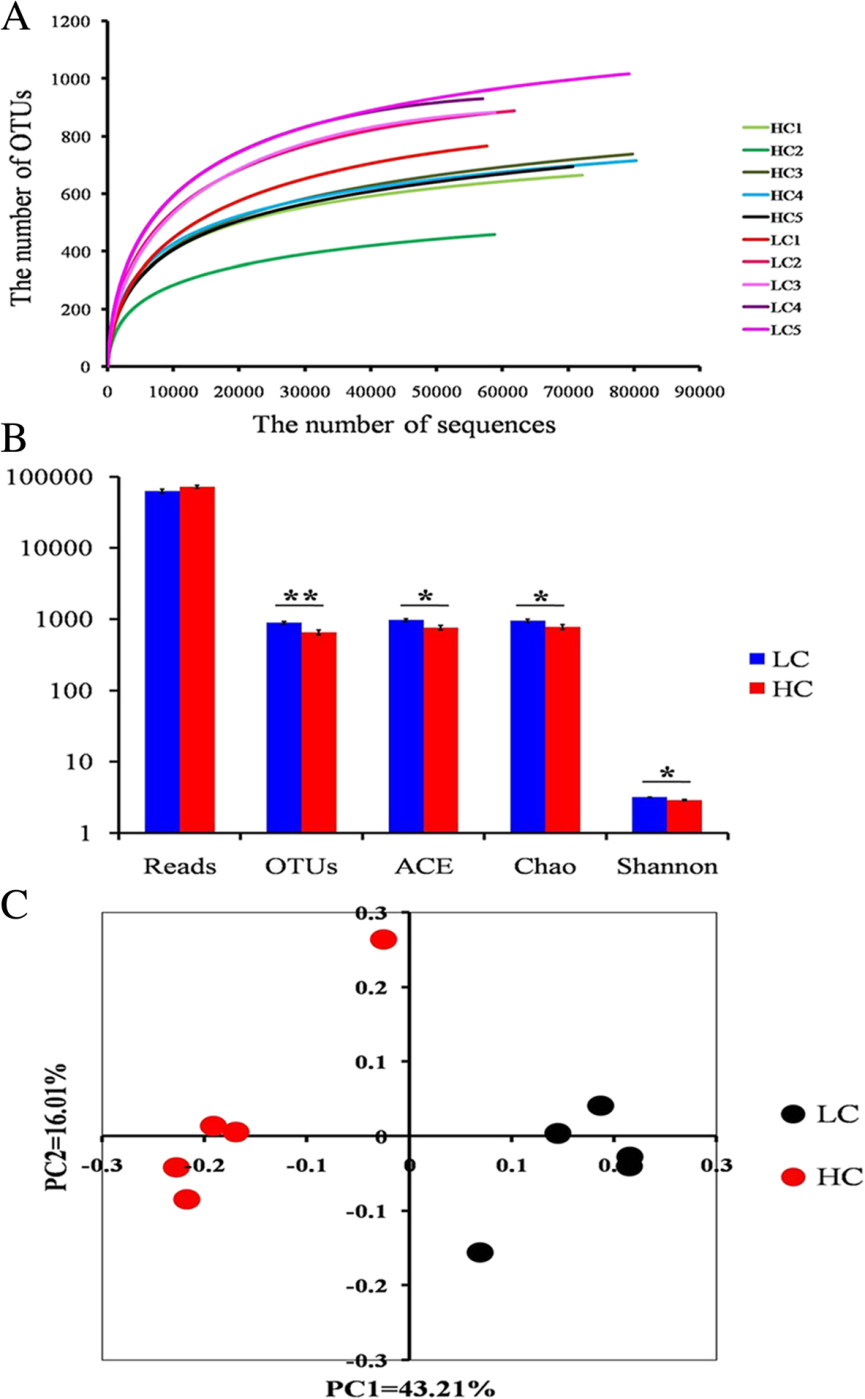
Pyrosequencing analysis of cecal microbiota of goats fed LC or HC diets for 10 weeks. **a** Rarefaction curves in the LC and HC groups. **b** Diversity indexes in the LC and HC groups (*n* = 5, mean ± SEM). OTU, operational taxonomic unit; ACE, abundance-based coverage estimator; Asterisks indicate the level of statistically significant difference: *P < 0.05; **P < 0.01. **c** Principal coordinate analysis (PcoA) based on the relative abundance of OTUs at a 97% similarity level in the LC or HC groups. PC1, principal coordinate 1; PC2, principal coordinate 2. LC, low concentration diet; HC high concentration diet

According to the pyrosequencing results, 355,543 qualified bacterial 16S rRNA gene reads were detected from 10 caecum content samples from the lactating goats and were applied for further analysis (Additional file 4: Table S4). The rarefaction analysis indicated that the HC diet decreased the numbers of operational taxonomic units (OTU) compared with the LC diet (Fig. 3a). At the taxonomic level, species richness was decreased in the HC group compared to that in the LC group, based on the analyses of the ACE and Chao1 indices (*P* < 0.05). Moreover, a significant difference (*P* < 0.05) was observed in the microbial community diversity between the LC group and the HC group, as reflected by the Shannon index (Fig. 3b and Additional file 5: Table S5). For the community compositions across all of the caecum content samples, the Bray-Curtis distance in mothur was used to assess the β-diversity. A principal coordinate analysis exhibited a distinct separation of the caecum content samples between the LC group and HC group (Fig. 3c).

**Fig. 4:**
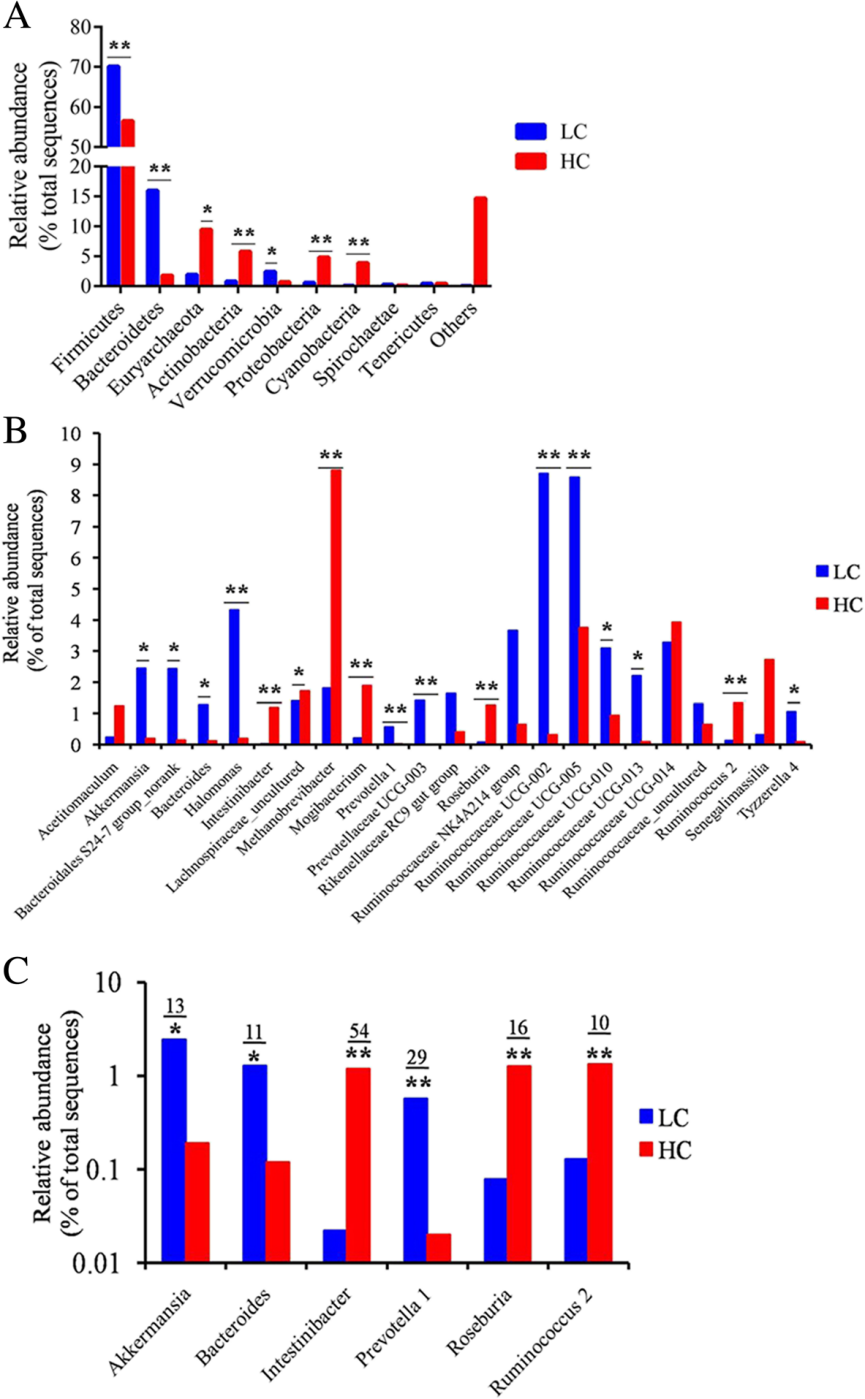
Effect of HC diet on the microbial communities at the phylum and genus levels. **a** The phylum comparison of bacterial OTUs between LC and HC groups (*n* = 5, values are medians). **b** The predominant genus (the relative abundance > 1% at least in one sample) comparison of bacterial OTUs between LC and HC groups (*n* = 5, values are medians). **c** The significantly changed genus (more than 10-fold changes of relative abundance) between LC and HC groups (*n* = 5, values are medians). Logarithmic coordinate axis was used in the panel A and C. Asterisks indicate the level of statistically significant difference: * *P* < 0.05; ** *P* < 0.01

At the phylum level, the distribution of bacterial phyla was displayed in Additional file 8: Figure S1. Sixteen bacterial phyla were identified in the caecum content of lactating goats fed the LC diet, and 18 phyla were identified in the caecum content of the lactating goats fed the HC diet (Additional file 4: Table S4). Firmicutes and Bacteroidetes were the two predominant phyla, contributing to 70.13 and 15.97% of intestinal microbiota in the LC group and to 56.57 and 1.78% in the HC group, respectively (Fig. 4a, Additional file 6: Table S6). The HC diet significantly decreased the abundance of Firmicutes (*P* < 0.01), Bacteroidetes (*P* < 0.01) and Verrucomicrobia (*P* < 0.05) and increased the abundance of Euryarchaeota (*P* < 0.05), Saccharibacteria (*P* < 0.01), Actinobacteria (*P* < 0.01) and Proteobacteria (*P* < 0.01) compared to the LC diet group.

At the genus level, the distribution of the predominant genera is shown in Additional file 9: Figure S2. A total of 195 bacterial genera were detected in the LC group, and 234 genera were detected in the HC group (Additional file 4: Table S4). The genera with a relative abundance greater than 0.5% (at least in one caecum content sample) are summarized and shown in Additional file 7: Table S7. Among these genera, the average relative abundance of 23 bacterial genera, in at least one group, was greater than 1%, and these values are presented in Fig. 4b. The statistical results of the genus levels showed that the abundances of *Akkermansia* (*P* < 0.05), *Bacteroides* (*P* < 0.05), *Halomonas* (*P* < 0.01), *prevotella 1* (*P* < 0.01)*, Prevotellaceae UCG-003* (*P* < 0.01), *Ruminococcaceae UCG-002* (*P* < 0.01), *Ruminococcaceae UCG-005* (*P* < 0.01)*, Ruminococcaceae UCG-010* (*P* < 0.05) and *Ruminococcaceae-013* (*P* < 0.05) were lower, whereas the abundances of *Intestinibacter* (*P* < 0.01), *Roseburia* (*P* < 0.01), *Methanobrevibacter* (*P* < 0.01)*, Mogibacterium* (*P* < 0.01), and *Ruminococcus 2* (*P* < 0.05) were higher in the caecum content of the HC diet-fed goats compared to the LC diet-fed goats (Fig. 4b). Moreover, the genera that revealed more than a 10-fold change in the relative abundance between the LC group and the HC group are shown in Fig. 4c. Compared with the LC diet, the HC diet increased the abundance of *Roseburia*, *Ruminococcus 2*, and *Intestinibacter* by 16-, 11-, and 54-fold, respectively, and decreased the abundance of *Akkermansia*, *Bacteroides*, and *prevotella 1* by 13-, 11-, and 29-fold, respectively*.* Interestingly, the abundance of *Candidatus saccharimonas* in the HC group was significantly elevated (*P* < 0.01) 293-fold compared to the LC group (Additional file 7: Table S7).

### SCFAs, LPS, starch and pH value in the caecum content

The total concentration of SCFAs in the HC group was higher (*P* < 0.01, Table 1) than that in the LC group. Compared with the LC diet, the HC diet significantly decreased the concentration of acetate (*P* < 0.05), whereas it increased the concentrations of propionate (*P* < 0.01), butyrate (*P* < 0.01) and valerate (*P* < 0.05) 2.84-fold, 2.39-fold, and 1.12-fold, respectively. The LPS concentration and starch concentration in the caecum samples increased (*P* < 0.01) 2.74-fold and 5.64-fold, respectively, in the HC group compared to the LC group. In addition, the caecum pH value was lower (*P* < 0.05) in the HC group than in the LC group.

**Table 1:** SCFAs, pH, Starch and LPS concentrations in cecal content of goats

Item	LC^a^	HC^a^	FC
Acetate(mM)	84.32 ± 1.27	56.43 ± 3.04*	1.49
Propionate(mM)	16.11 ± 1.84	45.75 ± 2.34**	2.84
Butyrate(mM)	5.85 ± 1.13	13.96 ± 2.23**	2.39
Isobutyrate(mM)	1.50 ± 0.04	1.65 ± 0.05	1.10
Valerate(mM)	1.70 ± 0.17	1.90 ± 0.11*	1.12
Isovalerate(mM)	0.96 ± 0.07	0.92 ± 0.08	0.96
Total concentration of SCFAs	110.44 ± 1.69	120.61 ± 2.51**	1.64
Starch (% of DM)	0.11 ± 0.03	0.62 ± 0.08**	5.64
LPS concentration (EU/mL)	7257.01 ± 1020.43	19,889.47 ± 2917.37**	2.74
pH Value	6.77 ± 0.05	5.68 ± 0.08*	–

^a^mean ± SE, *P < 0.05; **P < 0.01 vs LC group, *FC* Fold changes

### Correlation analyses between the caecum microbiome and SCFAs, starch or LPS

**Fig. 5:**
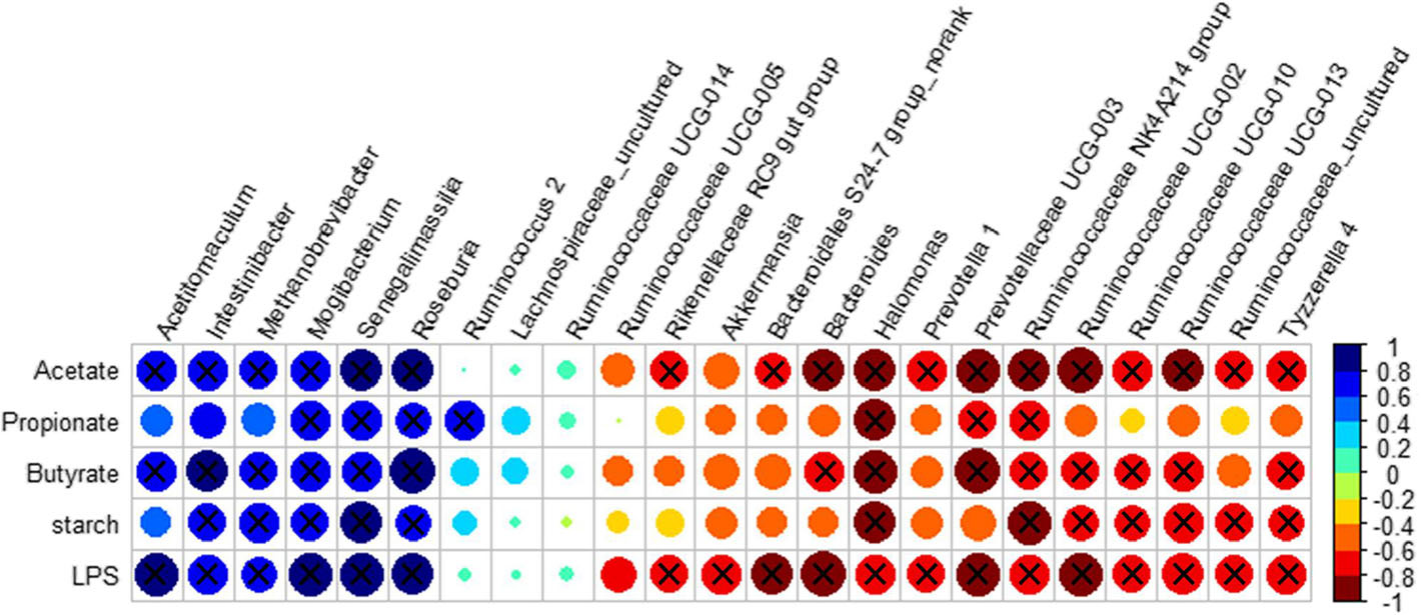
Correlation analysis between SCFAs, Starch, LPS and microbiota composition (at the genus level). Only the predominant bacterial genera (relative abundance ≥1% in at least one sample) was analyzed and shown. Circle’s size and color was drafted according to Spearman’s rank correlation coefficient between SCFAs, Starch, LPS and microbiota composition in the cecal content. **×** represents a significant correlation (*P* < 0.05)

A Spearman’s correlation analysis was carried out by calculating the Spearman’s rank correlation coefficient to explore the functional correlation between the caecal microbiome changes and the metabolite perturbations (Fig. 5). The correlation analysis revealed that acetate was positively associated (R > 0.6, *P* < 0.05) with 6 taxa and was negatively associated (R < − 0.6 and *P* < 0.05) with 12 taxa. Propionate was positively correlated (R > 0.6, *P* < 0.05) with 4 taxa and was negatively correlated (R < − 0.6 and *P* < 0.05) with 3 taxa. Butyrate was positively associated (R > 0.6, *P* < 0.05) with 6 taxa and was negatively associated (R < − 0.6 and *P* < 0.05) with 8 taxa. Moreover, our results also showed that starch was positively associated (R > 0.6, *P* < 0.05) with 5 taxa and was negatively associated (R < − 0.6 and *P* < 0.05) with 7 taxa. The LPS concentration was positively associated (R > 0.6, *P* < 0.05) with 6 taxa and was negatively associated 12 taxa.

### Expression of candidate genes in the caecal mucosa of lactating goats from the LC or HC groups

The expressions of mRNAs encoding G protein-coupled receptors (GPR41 and GPR43), cytokines (IL-1β, IL-6, TNF-α and IL-10), and chemokines (IL-8, CCL5 and CCL20) were increased in the caecal mucosa tissue of the lactating goats fed the HC diet compared to those fed the LC diet (Table 2). The concentrations of GPR41 and GPR43, which are specific receptors for SCFAs, were significantly increased 3.66- and 6.56-fold in the HC group compared with that in the LC group. However, TLR4 expression was not significantly different between the LC and HC groups. The HC diet increased the expressions of the pro-inflammatory cytokines IL-1β, IL-6, and TNF-α compared to the LC diet, and the fold increases differed between the LC and HC groups, and these differences are shown in Table 4. The expression of IL-10, an anti-inflammatory cytokine, was enhanced 2.83-fold in the HC group compared with the LC group. Enhanced expressions of the key chemokines IL-8, CCL5 and CCL20 were observed, with a 3.09-fold increase for IL-8, a 19.38-fold increase for CCL5 and a 4.97-fold increase for CCL20 in the HC group compared to the LC group.

**Table 2:** mRNA expressions [rel. Copy number] of candidate genes

Gene	Comment	LC^a^	HC^a^	FC
TLR4	LPS recognition receptor	943 ± 90	1083 ± 103	1.11
GPR41	G protein-coupled receptor	682 ± 103	2497 ± 217**	3.66
GPR43	G protein-coupled receptor	423 ± 78	2775 ± 274**	6.56
IL-1α	Cytokine	192 ± 40	544 ± 158	2.83
IL-1β	Cytokine	3015 ± 307	11,523 ± 1031*	3.82
IL-6	Cytokine	105 ± 71	2184 ± 315*	20.8
TNF-α	Cytokine	708 ± 91	4825 ± 173**	6.81
IL-8	Chemokine	841 ± 134	2597 ± 112*	3.09
IL-10	Cytokine	756 ± 81	2142 ± 412*	2.83
CCL5	Chemokine	160 ± 161	3052 ± 709*	19.38
CCL20	Chemokine	289 ± 86	1437 ± 287**	4.97

^a^mean + SE, *p < 0.05; **p < 0.01 vs LC group, *FC* fold changes

### Expression of target proteins in the caecal mucosa of lactating goats from the LC or HC groups

**Fig. 6:**
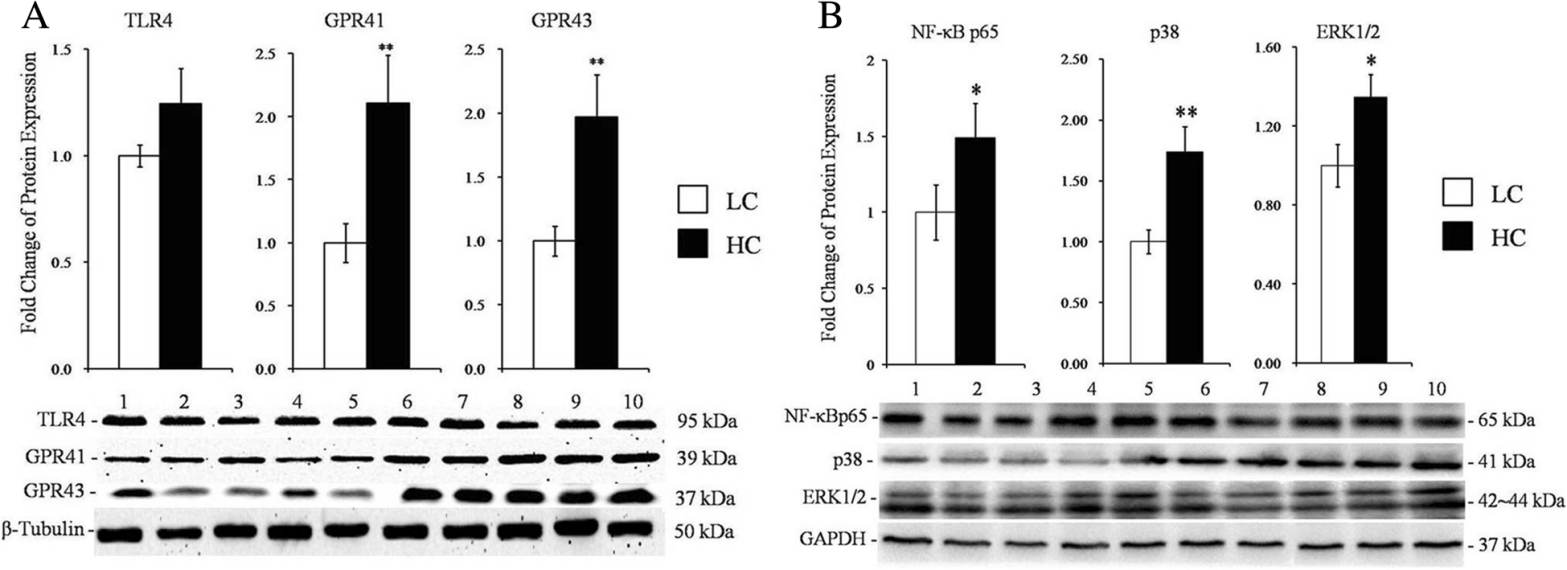
The protein expression of related receptors and transcriptional factors in the cecum mucosal tissue. **a** The protein levels of TLR4, GPR41 and GPR43 are expressed as arbitrary units relative to β-tubulin. **b** The protein levels of NF-κB, p38 and ERK1/2 are expressed as arbitrary units relative to GAPDH. Bands 1–5 represented the caecal mucosa samples from LC group; Bands 6–10 represented the caecal mucosa samples from HC group. Asterisks indicate the level of statistically significant difference: * *P* < 0.05; ** *P* < 0.01

Feeding the HC diet to lactating goats increased (*P* < 0.01, Fig. 6 and Additional file 10) the expression levels of GPR41 and GPR43 in the caecal mucosal tissue compared with the LC diet. However, TLR4 protein expression in the caecal mucosa tissue was not significantly different between the LC group and the HC group. Moreover, p38 protein expression was significantly enhanced (*P* < 0.01) in the HC group compared to the LC group, and the expression levels of NF-κB p65 and ERK1/2 in the HC group were higher (*P* < 0.05) than those in the LC group.

**Fig. 7:**
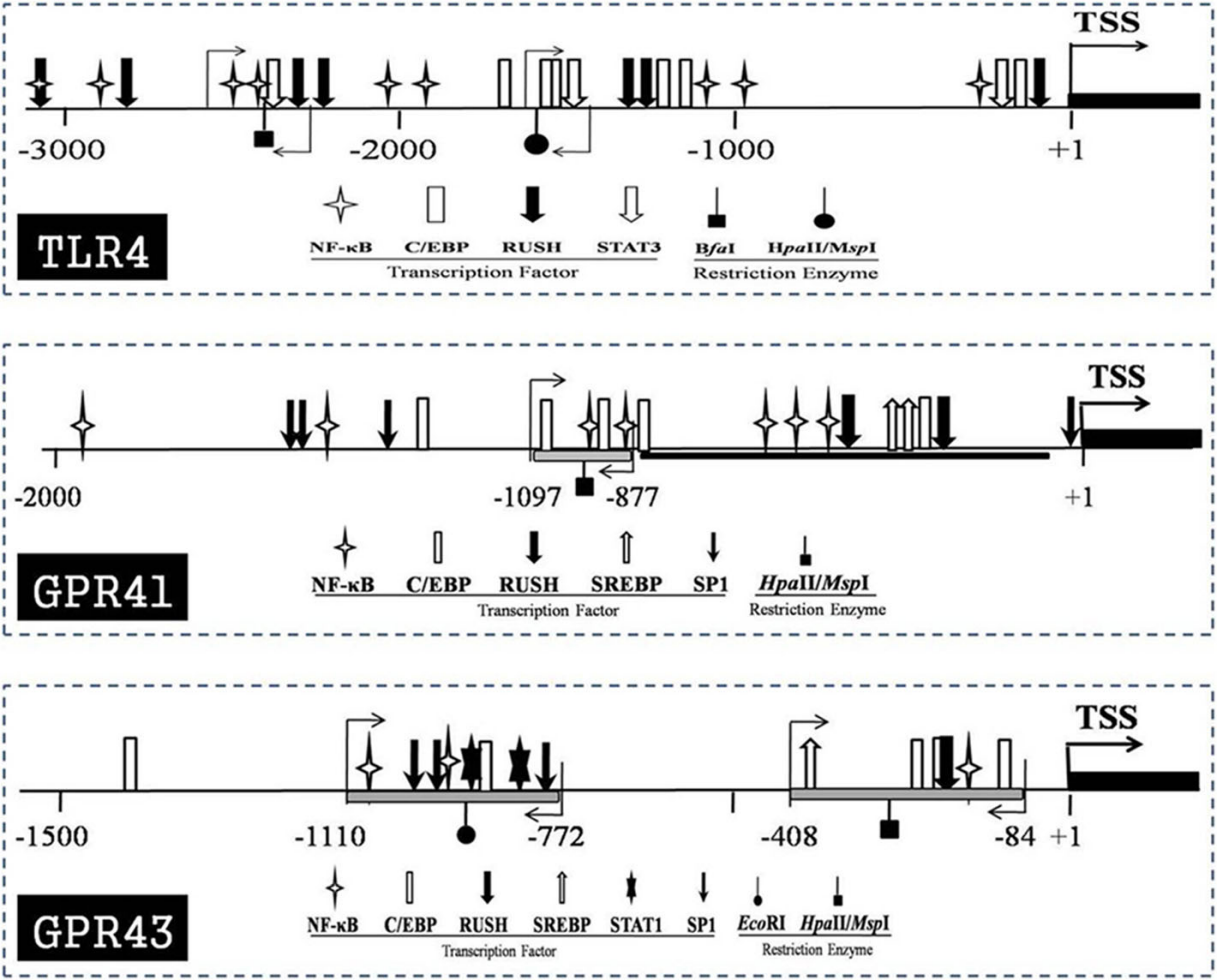
Map of binding site for the relevant transcriptional factors in the promoters of candidate genes. TSS indicates the transcriptional stats sites (+ 1), and the black filled box indicates the 5′-position of exon 1of target gene. The respective symbols are used to express the position s of various transcription factors and restriction enzyme recognition site. The positions of primers for CHART-PCR and methylation assay were denoted by the light arrows

The GPR41 and GPR43 promoters have not been thoroughly researched previously. Therefore, the DNA sequences of their promoters were identified via BLAST procedures using the mRNA sequences of GPR41 and GPR43 from the NCBI file NW_005100869.1. The MatInspector package (Genomatix, online tool) was used to analyse the potential binding sites for the relevant transcriptional factors and to verify the core promoter areas of GPR41 and GPR43. The promoter structure maps of GPR41 and GPR43 were prepared to exhibit the positions of the restriction enzymes and primer pairs involved in the assays of chromatin remodelling and DNA methylation (Fig. 7). The TLR4 promoter structure was described in detail in our previous study (24).

The chromatin accessibility by real-time PCR (CHART-PCR) results revealed that the HC diet significantly decreased (*P* < 0.05, Table 3) the average degree of the chromatin compaction at the promoter regions of GPR41 and GPR43 but did not affect TLR4 compared with the LC diet. Furthermore, the results of the DNA methylation assay indicated that the average degree of promoter methylation of GPR41 and GPR43 in the HC group was lower (*P* < 0.05, Table 4) than that in the LC group. However, the promoter methylation of TLR4 was not significantly different between the LC group and the HC group.

**Table 3:** The percentage of promoter compaction of candidate genes in cecal mucosa of goats

Gene (Compaction, %)	LC^a^	HC^a^
TLR4	0.74 ± 0.08	0.67 ± 0.05
GPR41	0.92 ± 0.04	0.21 ± 0.06^**^
GPR43	0.85 ± 0.04	0.17 ± 0.05^**^

^a^mean + SE, *p < 0.05; **p < 0.01 vs LC group

**Table 4:** The percentage of promoter methylation of candidate genes in cecal mucosa of goats

Gene (Methylation, %)	LC^a^	HC^a^
TLR4	0.46 ± 0.05	0.42 ± 0.04
GPR41	0.44 ± 0.04	0.12 ± 0.03^**^
GPR43	0.53 ± 0.06	0.13 ± 0.03^**^

^a^mean + SE, *p < 0.05; **p < 0.01 vs LC group

**Fig. 8:**
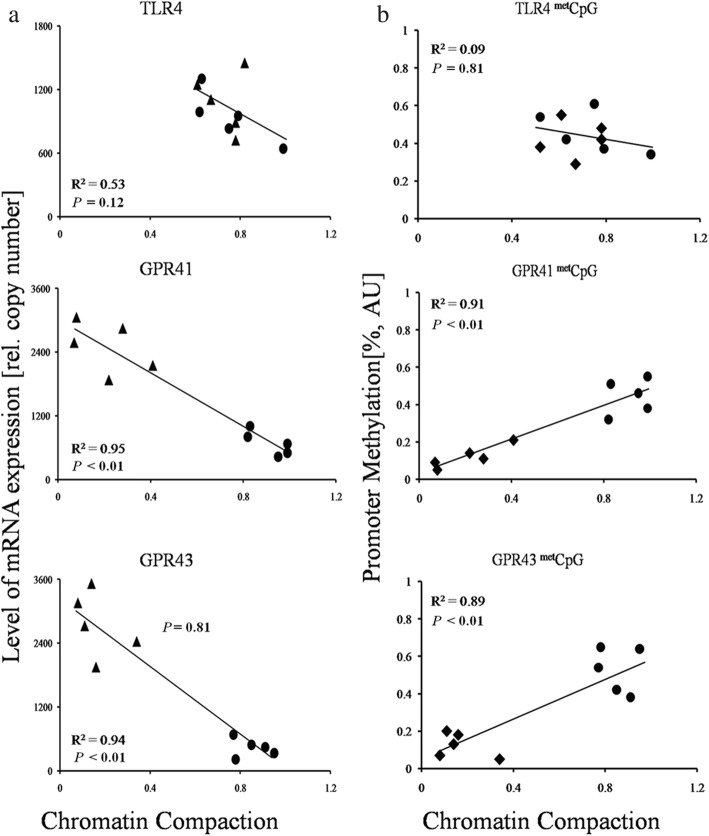
Correlation analysis between chromatin compaction and mRNA expression or DNA methylation. R, Pearson’s correlation coefficient; *p*, significance of correlation. AU, arbitrary units. **a** The relationship between mRNA expression and chromation compaction. **b** the relationship between DNA methylation and chromatin compaction

The results of the Pearson’s correlation analyses revealed a strong relationship between the degree of chromatin compaction and the relative mRNA expression for GPR41 and GPR43 (Fig. 8). The correlation coefficients for GPR41 (*r*^*2*^ = 0.95, *P* < 0.01) and GPR43 (*r*^*2*^ = 0.94, *P* < 0.01) were robust and significant. Additionally, the results of the correlation analyses between the chromatin compaction and DNA methylation showed a significant positive correlation in GPR41 (*r*^*2*^ = 0.91, *P* < 0.01) and GPR43 (*r*^*2*^ = 0.89, *P* < 0.01). However, no significant correlation between chromatin compaction and mRNA expression (*r*^*2*^ = 0.53, *P* = 0.12) or DNA methylation (*r*^*2*^ = 0.09, *P* = 0.81) was obtained for TLR4.

## Discussion

Mounting evidence suggests that the long-term consumption of an HC diet by lactating ruminants leads to SARA, which causes a diverse range of secondary diseases, including ruminitis, liver inflammation and abscesses, endometritis and diarrhoea [5, 18, 19]. Although several rumen pH thresholds (for example, 6.0, 5.8, and 5.6) have been used to define SARA, the most widely used definition is a rumen pH < 5.6 for at least 180 min/day [3, 4]. In the current study, our results showed that the duration of the rumen pH value being below 5.6 was more than 180 min/day from the 5th week onward in the HC group compared with the LC group. This finding indicated that SARA was successfully induced by feeding the HC diet to the lactating goats. In addition, we also observed that the HC diet declined the milk yield, milk fat, and milk protein after 10 weeks of feeding, and significant differences in the milk yield, milk fat, and milk protein were obtained at the 9th, 7th, and 10th weeks, respectively. These findings, consistent with a previous experimentally induced SARA study [2, 20], further indicated the occurrence of SARA in our present study.

The composition of the digestive tract microbiota is widely demonstrated to be closely related to dietary structure [1, 21]. In the present study, in line with previous reports [6, 22], the HC-induced SARA reduced the bacterial richness and diversity, as revealed by the ACE and Shannon indices. Meanwhile, the results from the PCOA exhibited a distinct difference in the composition of the caecum bacterial community between the LC and HC groups. These findings, combined with the decline in the caecum pH and the increase in the caecal starch content, indicated that SARA disrupted the ecological balance of the microbiota in the caecum of lactating goats. This disruption may be due to the increased caecum fermentable carbohydrate provided by the HC diet, promoting the amylolytic or other starch-digesting bacterial proliferation. Additionally, recent studies show a strong interplay between the gastrointestinal microbiota and the host immune and metabolic functions [23, 24]. Here, we observed that the abundance of two predominant phyla (*Firmicutes* and *Bacteroidetes*) in the caecal microbiota was significantly decreased with HC diet-induced SARA. The decline in *Bacteroidetes*, consistent with a previous study [6], contributed to the explanation about the increased LPS concentration in the caecal content of the lactating goats fed the HC diet [25, 26]. Furthermore, we also observed a significant negative correlation between the concentration of free LPS and the abundance of gram-negative bacteria, including the taxa *Akkermansia*, *Bacteroides*, *Halomonas*, *Prevotella 1* and *Prevotellaceae UCG-003*. These findings, combined with the 2.74-fold increased LPS concentration, revealed that HC diet-induced SARA altered the composition of the caecal microbiota, causing the death and lysis of gram-negative bacteria, thereby elevating the LPS concentration in the caecum of the lactating goats.

SCFAs, in particular acetate, propionate, and butyrate, are the major metabolites of the gut microbiota that use carbohydrates as substrates [27]. Our present work demonstrated that the total SCFAs in the caecum increased with HC diet-induced SARA. Interestingly, compared to the LC group, the concentrations of propionate and butyrate increased more than 2-fold, whereas the concentration of acetate decreased in the HC group. This finding was in accordance with the statistical results of the genera abundances and the correlation between the bacterial composition at the genus level and the SCFAs. *Ruminococcus* has been described previously as the major propionate-producing bacteria [27], and in the present study, *Ruminococcus 2* was significantly increased 10-fold in abundance in the HC group and exhibited a positive correlation with the concentration of propionate. The bacterial genus *Roseburia* increased 16-fold in abundance in the HC group, and it is considered a butyrate-producing bacteria in human intestinal content [28]. *Akkermansia*, *Bacteroides*, and *Prevotella 1*, which are considered the major bacterial genera that produce acetate [27], were markedly decreased 13-, 11-, 29-fold, respectively, in their abundance in the HC group. Meanwhile, *Bacteroides* and *Prevotella 1*, in terms of their abundance, were negatively associated with the concentration of acetate. Interestingly, *Intestinibacter* is also an acetate-producing bacterial genus, and its abundance increased 54-fold in the HC group and was positively correlated with the concentration of acetate. This result may contribute to explaining why SARA resulted in a slight decrease in the acetate concentration in the present study as well as that no significant difference in the acetate concentration was found between the HC group and LC group in a previous study [29]. In short, with HC diet-induced SARA, the alterations in the caecal microbiota significantly increased the concentration of propionate and butyrate and decreased the concentration of acetate in the caecal content of the lactating goats.

Our mRNA expression analysis distinctly verified that the lactating goats suffering from SARA were subjected to mucosal inflammation of the caecum. This finding was in line with the previous findings from lactating goats, in which SARA was caused by feeding of the HC diet [26]. These goats showed inflammatory injury based on the histology analysis in the caecal mucosa. However, although the LPS concentration was increased in the caecal content, the expression of TLR4, the specific receptor of LPS, was unchanged in the caecum mucosal tissue of these goats.

Previous studies focusing on human or ruminant diseases indicated that expression of TLR4 are involved in epigenetic modification [30, 31]. In the present study, our results showed that epigenetic alterations (chromatin remodelling and DNA methylation) in the TLR4 promoter were not elicited with HC diet-induced SARA. This finding further elucidated that the caecal inflammation was not caused by the activation of the TLR4 signalling pathway, which is one of the major TLR signalling pathways causing inflammation [32]. Moreover, five levels of evidence were identified in our present work to reveal the importance of GPR41 and GPR43 signalling in caecal inflammation. First, SCFAs are widely reported to be the ligands of the GPR41 and GPR43 receptors [33, 34], and we found that the total SCFA concentration was significantly increased, especially that of propionate and butyrate, in the caecal content of the lactating goats suffering from SARA. Second, our results illustrated that HC diet-induced SARA enhanced the expressions of GPR41 and GPR43 mRNA and protein in the caecum mucosal tissue. Third, p38 and ERK1/2, as the downstream molecules of the GPR41 and GPR43 signalling pathways, have been verified in previous studies [14, 35], and we observed an increase in p38 and ERK1/2 proteins in the caecum mucosal tissue in goats suffering from SARA. Fourth, a previous study showed that the induction of cytokines and chemokines by SCFAs in intestinal epithelial cells occurs in a GPR41- and GPR43-dependent manner, and a significant increase in the pro-inflammatory cytokines and chemokines in the caecum mucosal tissue was clearly observed in the present study. Fifth, it is well-known that the development of inflammation can be regulated by epigenetic modifications via promoting or depressing the expression of inflammation-related genes [36], and the depression of chromatin compaction and DNA methylation observed in the present study distinctly suggested that epigenetic mechanisms were involved in the regulation of GPR41 and GPR43 expression in the caecum mucosal tissue with SARA. Together, these diverse levels of evidence clearly demonstrated that the activation of the GPR41 and GPR43 signalling pathways by SCFAs lead to caecum mucosal inflammation, and epigenetic modifications enhanced the expression of GPR41 and GPR43 in lactating goats suffering from SARA.

## Conclusions

Collectively**,** our results indicated that HC diet-induced SARA altered the composition of the microbiota in the caecal content. On the one hand, although this alteration increased the concentration of LPS, the TLR4 signalling pathway, which is a specific receptor of LPS, was not activated. On the other hand, the shift in the microbiota elevated the concentration of SCFAs (especially propionate and butyrate). Subsequently, the activation of the GPR41 and GPR43 signalling pathways by the SCFAs increased the production of cytokines and chemokines and caused caecal inflammation, and epigenetic mechanisms contributed to the development of this inflammation in lactating goats suffering from SARA.

## Methods

### Animals, diets and experimental design

The experimental design and procedures were authorized by the Animal Care and Use Committee of Nanjing Agricultural University in compliance with the Regulations for the Administration of Affairs Concerning Experimental Animals (The State Science and Technology Commission of People’s Republic of China, 1988).

Ten multiparous lactating goats, with a body weight of approximately 32.43 ± 2.14 kg, were purchased from dairy goats farm (Yangling, Shanxi Province, China) and installed with rumen fistula and housed in individual metabolic cages. Four weeks before the beginning of the formal experiment, all the goats were administered a LC diet to manipulate the similarity of metabolic statuses. After the rumen surgery and dietary adaption, the goats were randomly separated into two groups and received either the LC diet (*n* = 5) or the HC diet to induce subacute ruminal acidosis (SARA). The goats were milked and administered an equal amount of diet (800 g dry matter per goat/day) at 0800 and 1630 daily for 10 weeks. The milk yield was recorded every day during the experimental period. Fresh water was freely available to all of the goats in this experiment. The details of the ingredients composition of both diets are summarized in Additional file 1: Table S1.

### Sample collection and determination of milk, SCFAs, starch and LPS

Rumen liquid (1 mL per time) was sampled at 30-min intervals for 24 h in the last 2 days of each week during the experimental period to measure the pH value with a basic pH metre (Sartorius, Goettingen, Germany). The milk (50 mL) sampled daily was preserved with potassium dichromate and was stored at 4 °C for determining the parameters of the milk fat and protein with a FossMatic 5000 analyser (FOSS Electric A/S, Denmark).

On the last day of this experiment, all of the goats were anaesthetised by intramuscular injection with Xylazine hydrochloride (0.8 mL per goat), and then executed by bloodletting through the carotid artery. After slaughtering, samples of caecum content and mucosa were obtained. The total caecum content of each goat was collected and mixed well; then, the pH value was measured immediately. Then, the well-mixed content was allocated into four portions (approximately 15 g wet weight per portion) for analyses of LPS, SCFAs, starch and microbial DNA sequencing. The detailed processing method of caecum content samples was described in previous study [6]. The total mucosa samples were divided into 2 cryogenic tubes (approximately 5 g per tube) and were immediately frozen in liquid nitrogen for RNA and DNA extractions.

The SCFA concentration was determined by gas chromatography using an FFAP 123–3233 capillary column (30 m × 0.32 mm × 0.5 μm, Agilent Technologies, Stevens Creek Blvd, Santa Clara, CA, USA) in an Agilent 7890A system (Agilent Technologies) [18]. The detection methods of starch LPS were described in the previous study in detail [6].

### Microbial DNA isolation and high-throughput pyrosequencing analysis

A total of 300 mg of caecal content from each goat was used to extract the total genomic DNA from the bacterium by using a bead-beating method and phenol-chloroform extraction [37]. The DNA concentration was measured by means of a Nanodrop 1000 (Thermo Fisher Scientific, Wilmington, DE, USA) and was stored at − 20 °C until further processing. The V3-V4 region of the bacterial 16S rRNA gene was amplified using a universal forward primer (5′- TACGGRAGGCAGCAG-3′) and a reverse primer (5′-AGGGTATCTAATCCT-3′). The amplicons purified using the AxyPrep DNA Gel Extraction Kit (Axygen Biosciences, Union City, CA, USA) were analysed on the Illumina MiSeq high through-put sequencing platform. The raw sequence data generated from the MiSeq sequencing were processed using the mothur software package (version 1.36.1, Department of Microbiology and Immunology, The University of Michigan), and the detailed procedures of analysis were described in the previous study [37], Rarefaction curves were constructed to quantify the coverage and sampling effort. Bacterial diversity was evaluated using the abundance-based coverage estimator (ACE), Chao 1 and Shannon indices. Principal coordinate analysis (PCoA) was conducted based on the Bray-Curtis distance. The difference in the sequence percentage between the LC and HC groups was evaluated using a nonparametric Mann-Whitney U test. The sequence information was submitted to GenBank under the accession number SRX3415167-SRX3415176.

### RNA extraction and gene expression

The total RNA was extracted with Trizol (Takara, Dalian, China) from ten caecal mucosa samples frozen in liquid nitrogen. cDNA was prepared by reverse transcription (cat. RR036A, Takara) from 1.5 μg of total RNA from each sample, and quantification of the mRNA copy numbers was done with the ABI 7300 instrument (Applied Biosystems, Foster City, CA, USA) and SYBR Green Kit (cat. DRR420A, Takara) as previously described [38]. the copy numbers of the detected mRNAs were calculated via the corresponding external standard curve, which was established using standard plasmid of each mRNA target sequence. The amplification primers are shown in Additional file 2: Table S2.

### Chromatin compaction determination

A detailed description of the chromatin preparation was shown in our previous study [38]. Chromatin compaction was evaluated using the CHART-PCR as previously described [39]. In the current study, chromatin compaction in the region of the promoters of TLR4, GPR41, and GPR43 was measured using the restriction enzymes B*fa*I, H*pa*II, and E*co*RI. The quantification of the undigested target DNA was amplified by real-time PCR (ABI 7300, USA), and the copy numbers were calculated against the corresponding external standard curve. The degree of compaction is shown as the percentage of the copy numbers determined from the digested vs undigested samples. The primers used to amplify the target promoter sequence of the candidate genes are listed in Additional file 2: Table S2.

### Methylation assay for TLR4, GPR41 and GPR43

Genomic DNA was isolated from the caecum mucosa using DNAzol Reagent (cat.10503027, Thermo Fisher, USA) according to the manufacturer’s protocol and was analysed by a Methyl-Profiler DNA Methylation Quantification of Real-Time PCR (qRT-PCR) assay [38]. The principles and procedures of this method were described in the previous study [38]. The degree of methylation of our candidate genes, TLR4, GPR41 and GPR43, is presented by the difference between the ratio of the H*pa*II- and M*sp*I-digested DNA. The primers for the methylation detection are listed in Additional file 2: Table S2.

### Western blotting

The total protein from each caecal mucosa sample was extracted with RIPA Lysis Buffer (cat.SN338, Sunshine Biotechnology Co., China). The concentration of the total protein was measured by the Pierce™ BCA Protein assay kit (cat.23225, Themo Fisher, USA). The detection metod of cadidate protein was indicated in the previous study [19]. The primary antibodies used in the current study included TLR4 (sc-293,072, Santa Cruz, USA), GPR41 (ab103718, Abcam, USA), GPR43 (ab131003, Abcam, USA), NF-κB (An365, Beyotime, China), p38 (ab31828, Abcam, USA), ERK1/2 (ab17942, Abcam, USA), β-tubulin (KC-5 T01, Kang Chen Bio-tech, China), and GAPDH (AP0066, Bioworld, USA). The expression level of each candidate protein is shown as the fold change of the average value between the LC and HC groups.

### Statistical analysis

The milk data were analysed as a repeated measure using the MIXED procedures of SAS (SAS version 9.4, SAS Institute Inc.). For the milk yield, milk protein, and milk fat, the effect of diet and week were considered as fixed factors, the effect of week was treated as a repeated measure, and the effect of the experimental animal was considered as a random factor. A non-parametric (Wilcoxon) method of SAS was used to explore the differences between the LC and HC groups for the microbial data (OTU level, phyla level and genera level). Correlations of the bacterial abundance (genus percentage from pyrosequencing analysis) with the SCFAs, starch, and LPS were analysed by using a Spearman’s correlation analysis of SAS, and correlations between the gene expression and chromatin compaction or DNA methylation in the area of the candidate gene promoter were calculated by using a Pearson’s correlation analysis of SAS. For the rest of the data (protein expression, mRNA expression, LPS, SCFAs, starch, chromatin compaction and DNA methylation), an analysis of variance (ANOVA) of SAS was used to evaluate the differences between the LC and HC groups. The differences were considered significant at *P* < 0.05 and were highly significant at *P* < 0.01.

## Additional files

Additional file 1: **Table S1.** Chemical composition and nutrient level of diets. (DOCX 14 kb)

Additional file 2: **Table S2.** The primers list for mRNA expression, chromatin compaction and DNA methylation. (DOCX 14 kb)

Additional file 3: **Table S3.** Rumen pH value, milk yield and milk components of the goats from LC and HC group. (DOCX 13 kb)

Additional file 4: **Table S4.** Summary of sequences, phyla and genus of caecal content samples from LC group or HC group. (DOCX 13 kb)

Additional file 5: **Table S5.** The phylogeny and relative abundance of the OTUs (abundance > 0.1% in one sample at least) in cecal content of lactating goats from LC or HC group. (DOCX 95 kb)

Additional file 6: **Table S6.** Relative abundance of bacterial phyla in cecal content of goats from LC group or HC group. (DOCX 14 kb)

Additional file 7: **Table S7.** Relative abundance of bacterial genera in cecal content of lactating goats from LC or HC group. (DOCX 22 kb)

Additional file 8: **Figure S1.** The distribution of all bacterial phyla in the contents of cecum observed between LC group and HC group. LC1-LC5, the cecal content samples from goat fed low concentration diet; HC1-HC5, the cecal content samples from goats fed high concentration diet. (JPG 1308 kb)

Additional file 9: **Figure S2.** The distribution of the genera in the caecal contens of goats from LC group or HC group. Only genera which average relative abundance were greater than 0.5% and were significant difference (*p* < 0.05) between LC and HC group, were displayed. (JPG 1237 kb)

Additional file 10: Western blotting image pictures. (DOCX 886 kb)

## Data Availability

All the data supporting our findings is contained within the manuscript. And the raw sequence data were submitted to GenBank under the accession number SRX3415167-SRX3415176.

## Abbreviations

GPRs: G-protein–coupled receptors
HC: High concentrate
LC: Low concentrate
LPS: Lipopolisaccharide
OTU: Operational taxonnomic unit
PcoA: Principal component analysis
SARA: Subacute ruminal acidosis
SCFAs: Short chain fate acids
