# Supplementary material for: Microbial community shifts elicit inflammation in the caecal mucosa via the GPR41/43 signalling pathway during subacute ruminal acidosis
Source: BMC Vet Res. 2019 Aug 19;15:298. doi: 10.1186/s12917-019-2031-5 (PMC6700796; doi:10.1186/s12917-019-2031-5)
Supplement: Supplementary file 1 — Table S1. Chemical composition and nutrient level of diets. (DOCX 14 kb) [file 12917_2019_2031_MOESM1_ESM.docx]

Table S1. Chemical composition and nutrient level of diets

| （Ingredient） | | Percentage (%)of ingredients in different diets (dry matter) | |
| --- | --- | --- | --- |
|  |  | LC diet | HCdiet |
| Chinese wildrye hay | | 40.00 | 26.70 |
| Alfalfa hay | | 20.00 | 13.30 |
| Corn | | 22.99 | 23.24 |
| Bran | | 0 | 20.77 |
| Soybean meal | | 15.00 | 13.66 |
| limestone | | 0.65 | 1.43 |
| Calcium phosphate dibasic | | 0.46 | 0.00 |
| Salt | | 0.4 | 0.40 |
| Premix ^a^ | | 0.5 | 0.50 |
| Forage : Concentrate(F:C) | | 6:4 | 4:6 |
| **Nutrient level, % of dry matter** | | | |
| Net energy, MJ/kg | 5.73 | | 5.83 |
| DCP, % | 9.90 | | 10.00 |
| NDF, % | 36.64 | | 34.55 |
| ADF, % | 24.74 | | 20.35 |
| NFC，% | 31.76 | | 35.00 |
| Ca，% | 0.80 | | 0.90 |
| P,% | 0.33 | | 0.38 |

^a^ Premix provided： 3000, 1250, and 40 IU kg^-1^ of diet of vitamin A, D and E, and 6.25, 62.5, 62.5, 50, 0.25, 0.125, 0.125 mg kg^-1^ of diet of Cu, Fe, Zn, Mn, I, Se, Co, respectively. LC, low concentration; HC, High concentration.
