# Supplementary material for: Microbial community shifts elicit inflammation in the caecal mucosa via the GPR41/43 signalling pathway during subacute ruminal acidosis
Source: BMC Vet Res. 2019 Aug 19;15:298. doi: 10.1186/s12917-019-2031-5 (PMC6700796; doi:10.1186/s12917-019-2031-5)
Supplement: Supplementary file 2 — Table S2. The primers list for mRNA expression, chromatin compaction and DNA methylation. (DOCX 14 kb) [file 12917_2019_2031_MOESM2_ESM.docx]

Table S2. The primers list for mRNA expression, chromatin compaction and DNA methylation*.*

| Gene | Forward primer | Reverse primer | Comment |
| --- | --- | --- | --- |
| TLR4 | CTGAGAACCGAGAGCTGGGAC | TTCGCATCTGGATAAATCCAGC | mRNA |
| GPR41 | CGGACTTGATCTGGAGGAGC | GTGAAGAAGAGGAACCTGGAG | mRNA |
| GPR43 | GGAGAGGTGGAGAAGAATTTG | CAGACGTGGCTTCGATGATC | mRNA |
| IL-1α | GATGATGACCTGGAAGCCATTG | GCTGAGAATCCTCTTCTGATAC | mRNA |
| IL-1β | CCGTGATGATGACCTGAGGAG | CAAGACAGGTATAGATTCTTGTC | mRNA |
| IL-6 | CGAAGCTCTCATTAAGCACATC | CCAGGTATATCTGATACTCCAG | mRNA |
| TNF-α | CAACAGGCCTCTGGTTCAGAC | GGACCTGCGAGTAGATGAGG | mRNA |
| IL-8 | CTGAGAGTTATTGAGAGTGGGC | CAGTACTCAAGGCACTGAAGTAG | mRNA |
| IL-10 | GTGATGCCACAGGCTGAGAAC | GAAGATGTCAAACTCACTCATGG | mRNA |
| CCL5 | CTACACCAGCAGCAAGTGCT | CAAGCTGCTTAGGACAAGAGG | mRNA |
| CCL20 | GAAGCAGCAAGCAGCTTTGAC | GTTCCATTCCAGGGAGCATC | mRNA |
|  |  |  |  |
| TLR4 | CATAACAGCACTTCAAGGTAC | GGAAGCTGCTATGCATTAGAT | chromatin compaction |
| GPR41 | CTGTCTCTACAGATCTGCCTAC | CCGAACATTACCTGGTGTCCATC | chromatin compaction |
| GPR43 | GTTTCCTCAGCTGTGTGTTTCAG | CATGTAATGGATGCCCGTAAG | chromatin compaction |
|  |  |  |  |
| TLR4 | GCTTTGTCTATGCAGTCACTT | CAGTCCCTGCTCCAGGAAGAT | DNA methylation |
| GPR41 | CTGTCTCTACAGATCTGCCTAC | CCGAACATTACCTGGTGTCCATC | DNA methylation |
| GPR43 | GCATTTCACTTCACTTCACCACG | CCACTCGCCCAGAGATCCTCAC | DNA methylation |
