# Supplementary material for: Microbial community shifts elicit inflammation in the caecal mucosa via the GPR41/43 signalling pathway during subacute ruminal acidosis
Source: BMC Vet Res. 2019 Aug 19;15:298. doi: 10.1186/s12917-019-2031-5 (PMC6700796; doi:10.1186/s12917-019-2031-5)
Supplement: Supplementary file 3 — Table S3. Rumen pH value, milk yield and milk components of the goats from LC and HC group. (DOCX 13 kb) [file 12917_2019_2031_MOESM3_ESM.docx]

Table S3**.** Rumen pH value, milk yield and milk components of the goats from LC and HC group

| Item | LC^a^ | HC^a^ | *p* value | | |
| --- | --- | --- | --- | --- | --- |
|  |  |  | diet | week | diet × week |
| Rumen pH value | 6.45 ± 0.05 | 5.91 ± 0.11 | <0.01 | 0.03 | 0.15 |
| Milk yield (Kg) | 1.34 ± 0.04 | 1.23 ± 0.05 | 0.04 | < 0.01 | 0.08 |
| Milk protein (%) | 4.16 ± 0.07 | 4.11 ± 0.04 | 0.78 | 0.74 | 0.65 |
| Milk fat (%) | 3.39 ± 0.09 | 3.23 ± 0.09 | 0.44 | 0.58 | 0.88 |

^a^ mean ± SE; LC, low concentration; HC, high concentration.
