# Supplementary material for: Microbial community shifts elicit inflammation in the caecal mucosa via the GPR41/43 signalling pathway during subacute ruminal acidosis
Source: BMC Vet Res. 2019 Aug 19;15:298. doi: 10.1186/s12917-019-2031-5 (PMC6700796; doi:10.1186/s12917-019-2031-5)
Supplement: Supplementary file 4 — Table S4. Summary of sequences, phyla and genus of caecal content samples from LC group or HC group. (DOCX 13 kb) [file 12917_2019_2031_MOESM4_ESM.docx]

Table S4. Summary of sequences, phyla and genus of caecal content samples from LC group or HC group

| Item | LC | HC |
| --- | --- | --- |
| Total Sequences | 219625 | 135918 |
| Valid sequences | 200030 | 125561 |
| Phylum | 16 | 18 |
| Genus | 195 | 234 |

LC, low concentration; HC, high concentration.
