# Supplementary material for: Microbial community shifts elicit inflammation in the caecal mucosa via the GPR41/43 signalling pathway during subacute ruminal acidosis
Source: BMC Vet Res. 2019 Aug 19;15:298. doi: 10.1186/s12917-019-2031-5 (PMC6700796; doi:10.1186/s12917-019-2031-5)
Supplement: Supplementary file 5 — Table S5. The phylogeny and relative abundance of the OTUs (abundance > 0.1% in one sample at least) in cecal content of lactating goats from LC or HC group. (DOCX 95 kb) [file 12917_2019_2031_MOESM5_ESM.docx]

Table S5**.** The phylogeny and relative abundance of the OTUs (abundance >0.1% in one sample at least) in cecal content of lactating goats from LC or HC group*.*

| OTU ID | LC^a^ | HC^a^ | *p* value | type | phylum | family | Genus |
| --- | --- | --- | --- | --- | --- | --- | --- |
| OTU2 | 8.49(13.48, 3.38) | 0.22(1.32, 0.01) | < 0.01 | shared | Firmicutes | Ruminococcaceae | Ruminococcaceae UCG-002 |
| OTU3 | 0.20(0.53, 0.11) | 4.27(7.24, 1.12) | < 0.01 | shared | Proteobacteria | Halomonadaceae | Halomonas |
| OTU4 | 1.17(12.45, 0.01) | 0.56(2.21, 0.08) | 0.46 | shared | Verrucomicrobia | Verrucomicrobiaceae | Akkermansia |
| OTU5 | 1.66(10.06, 0.28) | 5.22(24.71, 2.32) | 0.05 | shared | Euryarchaeota | Methanobacteriaceae | Methanobrevibacter |
| OTU6 | 1.49(2.75, 0.16) | 0.07(0.87, 0.00) | 0.02 | shared | Firmicutes | Christensenellaceae | Christensenellaceae R-7 group |
| OTU7 | 3.18(4.27, 1.08) | 0.44(0.75, 0.02) | < 0.01 | shared | Firmicutes | Ruminococcaceae | Ruminococcaceae NK4A214 group |
| OTU8 | 1.19(14.52, 0.33) | 0.01(0.02, 0.00) | < 0.01 | shared | Firmicutes | Christensenellaceae | Christensenellaceae R-7 group |
| OTU9 | 0.03(16.77, 0.00) | 0.02(0.29, 0.01) | 0.92 | shared | Firmicutes | Christensenellaceae | Christensenellaceae R-7 group |
| OTU10 | 0.02(0.29, 0.01) | 0.81(6.11, 0.28) | 0.02 | shared | Firmicutes | Peptostreptococcaceae | Intestinibacter |
| OTU11 | 0.41(2.21, 0.17) | 1.03(1.42, 0.28) | 0.75 | shared | Firmicutes | Ruminococcaceae | [Eubacterium] coprostanoligenes group |
| OTU12 | 0.01(0.02, 0.00) | 1.60(4.53, 0.07) | < 0.01 | shared | Firmicutes | Family XIII | [Eubacterium] nodatum group |
| OTU13 | 2.04(5.55, 0.83) | 1.18(5.39, 0.10) | 0.25 | shared | Firmicutes | Ruminococcaceae | Ruminococcaceae UCG-005 |
| OTU14 | 0.00(0.15, 0.00) | 0.03(6.03, 0.00) | 0.17 | shared | Firmicutes | Ruminococcaceae | Ruminococcaceae UCG-014 |
| OTU15 | 0.68(2.16, 0.05) | 0.04(6.05, 0.01) | 0.12 | shared | Firmicutes | Lachnospiraceae | Pacaense |
| OTU16 | 0.05(0.09, 0.00) | 0.74(1.57, 0.12) | < 0.01 | shared | Actinobacteria | Coriobacteriaceae | Senegalimassilia |
| OTU17 | 0.01(0.14, 0.00) | 4.62(6.38, 0.98) | < 0.01 | shared | Saccharibacteria | Unknown Family | Candidatus Saccharimonas |
| OTU18 | 0.01(0.21, 0.00) | 0.57(5.51, 0.24) | < 0.01 | shared | Firmicutes | Peptostreptococcaceae | Intestinibacter |
| OTU19 | 0.76(5.83, 0.10) | 0.01(0.04, 0.00) | < 0.01 | shared | Firmicutes | Christensenellaceae | Christensenellaceae R-7 group |
| OTU20 | 0.15(0.33, 0.01) | 1.04(2.14, 0.49) | < 0.01 | shared | Firmicutes | Family XIII | Mogibacterium |
| OTU21 | 0.00(0.00, 0.00) | 0.23(5.01, 0.05) | < 0.01 | C.unique | Actinobacteria | Coriobacteriaceae | Senegalimassilia |
| OTU22 | 0.05(4.18, 0.00) | 0.02(0.07, 0.00) | 0.6 | shared | Firmicutes | Ruminococcaceae | Ruminococcaceae UCG-010 |
| OTU23 | 0.00(0.00, 0.00) | 0.04(5.32, 0.00) | 0.05 | C.unique | Actinobacteria | Bifidobacteriaceae | Bifidobacterium |
| OTU24 | 0.00(0.27, 0.00) | 0.16(3.42, 0.01) | 0.04 | shared | Firmicutes | Christensenellaceae | Christensenellaceae R-7 group |
| OTU25 | 0.02(0.17, 0.00) | 0.97(1.31, 0.21) | < 0.01 | shared | Firmicutes | Lachnospiraceae | Roseburia |
| OTU26 | 0.02(0.06, 0.00) | 0.26(3.72, 0.14) | < 0.01 | shared | Euryarchaeota | Methanobacteriaceae | Methanobrevibacter |
| OTU27 | 0.02(0.15, 0.00) | 0.64(3.75, 0.04) | 0.02 | shared | Firmicutes | Erysipelotrichaceae | Turicibacter |
| OTU28 | 0.39(1.18, 0.25) | 0.01(0.02, 0.00) | < 0.01 | shared | Firmicutes | Lachnospiraceae | Lachnospiraceae AC2044 group |
| OTU29 | 0.67(1.02, 0.15) | 0.64(1.34, 0.08) | 0.75 | shared | Firmicutes | Christensenellaceae | Christensenellaceae R-7 group |
| OTU30 | 0.04(1.13, 0.00) | 0.00(0.06, 0.00) | 0.09 | shared | Euryarchaeota | Methanocorpusculaceae | Methanocorpusculum |
| OTU32 | 0.47(2.98, 0.02) | 0.14(1.00, 0.01) | 0.6 | shared | Firmicutes | Ruminococcaceae | Ruminococcaceae UCG-014 |
| OTU33 | 0.00(0.02, 0.00) | 0.01(3.28, 0.00) | 0.45 | shared | Firmicutes | Family XIII | Family XIII AD3011 group |
| OTU34 | 0.00(0.05, 0.00) | 0.31(2.05, 0.03) | 0.01 | shared | Firmicutes | Clostridiaceae 1 | Clostridium sensu stricto 1 |
| OTU35 | 1.06(2.74, 0.17) | 0.10(0.20, 0.05) | 0.02 | shared | Firmicutes | Lachnospiraceae | Tyzzerella 4 |
| OTU36 | 0.03(2.49, 0.00) | 0.00(0.00, 0.00) | 0.02 | shared | Firmicutes | Ruminococcaceae | [Eubacterium] coprostanoligenes group |
| OTU37 | 0.00(0.01, 0.00) | 0.40(0.71, 0.24) | < 0.01 | shared | Euryarchaeota | Methanobacteriaceae | Methanosphaera |
| OTU38 | 0.00(0.01, 0.00) | 2.30(2.68, 0.32) | < 0.01 | shared | Saccharibacteria | Unknown Family | Candidatus Saccharimonas |
| OTU39 | 0.05(3.46, 0.01) | 0.03(0.10, 0.00) | 0.6 | shared | Firmicutes | Ruminococcaceae | Anaeromassilib |
| OTU40 | 0.05(2.24, 0.00) | 0.00(0.03, 0.00) | 0.07 | shared | Verrucomicrobia | Verrucomicrobiaceae | Akkermansia |
| OTU41 | 0.05(0.07, 0.03) | 0.59(0.89, 0.35) | < 0.01 | shared | Firmicutes | Family XIII | Family XIII AD3011 group |
| OTU42 | 0.05(0.19, 0.01) | 1.96(4.29, 1.16) | < 0.01 | shared | Firmicutes | Family XIII | Family XIII AD3011 group |
| OTU43 | 0.50(0.56, 0.09) | 0.00(0.01, 0.00) | < 0.01 | shared | Firmicutes | Ruminococcaceae | [Eubacterium] coprostanoligenes group |
| OTU44 | 0.14(2.38, 0.00) | 0.01(0.07, 0.00) | 0.33 | shared | Bacteroidetes | Porphyromonadaceae | Parabacteroides |
| OTU45 | 0.00(4.49, 0.00) | 0.00(0.01, 0.00) | 0.81 | shared | Bacteroidetes | Prevotellaceae | Prevotella 1 |
| OTU46 | 0.17(0.61, 0.05) | 0.18(0.32, 0.05) | 0.92 | shared | Firmicutes | Family XIII | Family XIII AD3011 group |
| OTU47 | 0.07(0.27, 0.00) | 0.14(1.64, 0.01) | 0.25 | shared | Firmicutes | Lachnospiraceae | Acetitomaculum |
| OTU48 | 0.02(0.18, 0.00) | 0.45(0.71, 0.21) | < 0.01 | shared | Firmicutes | Lachnospiraceae | Lachnospiraceae FE2018 group |
| OTU49 | 0.19(1.58, 0.11) | 0.05(0.15, 0.00) | 0.02 | shared | Firmicutes | Ruminococcaceae | Candidatus Soleaferrea |
| OTU50 | 0.02(0.11, 0.00) | 0.41(4.59, 0.15) | < 0.01 | shared | Firmicutes | Lachnospiraceae | Lachnospiraceae NK3A20 group |
| OTU51 | 0.00(0.00, 0.00) | 0.24(2.49, 0.00) | 0.03 | C.unique | Saccharibacteria | Unknown Family | Candidatus Saccharimonas |
| OTU52 | 0.00(0.15, 0.00) | 0.01(2.64, 0.00) | 0.59 | shared | Actinobacteria | Bifidobacteriaceae | Pseudocatenulatum |
| OTU53 | 0.47(2.03, 0.15) | 0.01(0.28, 0.00) | 0.05 | shared | Verrucomicrobia | Verrucomicrobiaceae | Akkermansia |
| OTU54 | 0.03(0.35, 0.01) | 0.48(1.54, 0.20) | 0.03 | shared | Actinobacteria | Coriobacteriaceae | Senegalimassilia |
| OTU55 | 1.53(3.17, 0.01) | 0.04(0.22, 0.00) | 0.12 | shared | Bacteroidetes | Rikenellaceae | Rikenellaceae RC9 gut group |
| OTU56 | 0.01(0.05, 0.00) | 0.21(1.31, 0.07) | < 0.01 | shared | Actinobacteria | Coriobacteriaceae | Atopobium |
| OTU57 | 0.00(0.01, 0.00) | 0.61(1.24, 0.23) | < 0.01 | shared | Saccharibacteria | Unknown Family | Candidatus Saccharimonas |
| OTU58 | 0.09(0.19, 0.00) | 0.29(0.80, 0.13) | 0.03 | shared | Firmicutes | Lachnospiraceae | Mobilitalea |
| OTU59 | 1.61(2.05, 0.36) | 0.36(0.67, 0.24) | 0.03 | shared | Firmicutes | Christensenellaceae | Christensenellaceae R-7 group |
| OTU60 | 0.00(0.98, 0.00) | 0.08(0.19, 0.00) | 0.75 | shared | Bacteroidetes | Flavobacteriaceae | Solitalea |
| OTU61 | 0.10(3.85, 0.01) | 0.08(0.19, 0.00) | 0.35 | shared | Bacteroidetes | Bacteroidaceae | Bacteroides |
| OTU62 | 0.16(0.66, 0.05) | 0.00(0.01, 0.00) | < 0.01 | shared | Firmicutes | Ruminococcaceae | Ruminococcaceae NK4A214 group |
| OTU63 | 0.12(0.74, 0.00) | 0.23(1.45, 0.01) | 0.92 | shared | Firmicutes | Ruminococcaceae | Saccharofermentans |
| OTU64 | 0.60(2.08, 0.14) | 0.02(0.05, 0.00) | < 0.01 | shared | Firmicutes | Ruminococcaceae | [Eubacterium] coprostanoligenes group |
| OTU65 | 0.03(2.86, 0.00) | 0.00(0.00, 0.00) | 0.05 | H.unique | Bacteroidetes | Bacteroidales BS11 gut group | Barnesiella |
| OTU66 | 0.22(1.42, 0.04) | 0.00(0.01, 0.00) | < 0.01 | shared | Bacteroidetes | Prevotellaceae | Prevotellaceae UCG-003 |
| OTU67 | 0.00(2.03, 0.00) | 0.02(0.44, 0.00) | 0.46 | shared | Bacteroidetes | Bacteroidales S24-7 group | Muribaculum |
| OTU68 | 0.22(0.58, 0.01) | 0.19(0.47, 0.13) | 0.75 | shared | Firmicutes | Ruminococcaceae | Ruminococcaceae UCG-014 |
| OTU70 | 0.53(2.69, 0.12) | 0.01(0.04, 0.00) | < 0.01 | shared | Bacteroidetes | Bacteroidaceae | Bacteroides |
| OTU71 | 0.91(2.38, 0.41) | 0.54(0.60, 0.02) | 0.17 | shared | Firmicutes | Ruminococcaceae | Ruminococcaceae UCG-005 |
| OTU72 | 0.48(1.74, 0.22) | 0.07(0.24, 0.00) | 0.02 | shared | Firmicutes | Ruminococcaceae | [Eubacterium] coprostanoligenes group |
| OTU74 | 0.00(3.35, 0.00) | 0.00(0.00, 0.00) | 0.14 | H.unique | Bacteroidetes | Bacteroidales S24-7 group | Muribaculum |
| OTU75 | 0.06(0.12, 0.06) | 0.00(0.01, 0.00) | < 0.01 | shared | Firmicutes | Lachnospiraceae | Lachnospiraceae FCS020 group |
| OTU77 | 0.00(1.12, 0.00) | 0.00(0.00, 0.00) | 0.14 | H.unique | Bacteroidetes | Prevotellaceae | Prevotellaceae UCG-003 |
| OTU78 | 0.24(0.58, 0.00) | 0.00(0.04, 0.00) | 0.17 | shared | Firmicutes | Ruminococcaceae | Ruminococcaceae UCG-010 |
| OTU79 | 0.00(1.01, 0.00) | 0.07(0.28, 0.02) | 0.12 | shared | Saccharibacteria | Unknown Family | Candidatus Saccharimonas |
| OTU80 | 0.00(0.00, 0.00) | 0.14(1.03, 0.00) | < 0.01 | C.unique | Saccharibacteria | Unknown Family | Candidatus Saccharimonas |
| OTU81 | 0.00(0.02, 0.00) | 0.32(0.63, 0.10) | < 0.01 | shared | Firmicutes | Family XIII | [Eubacterium] brachy group |
| OTU83 | 0.01(0.04, 0.00) | 0.02(1.38, 0.00) | 0.53 | shared | Euryarchaeota | Methanobacteriaceae | Methanobrevibacter |
| OTU84 | 0.05(1.26, 0.00) | 0.01(0.05, 0.00) | 0.45 | shared | Bacteroidetes | Bacteroidaceae | Bacteroides |
| OTU85 | 0.11(0.46, 0.00) | 0.23(0.75, 0.12) | 0.17 | shared | Firmicutes | Lachnospiraceae | [Ruminococcus] gnavus |
| OTU87 | 0.00(0.91, 0.00) | 0.49(1.91, 0.00) | 0.12 | shared | Firmicutes | Christensenellaceae | Christensenellaceae R-7 group |
| OTU88 | 0.13(0.57, 0.06) | 0.03(0.11, 0.00) | 0.03 | shared | Firmicutes | Ruminococcaceae | Ruminococcus 2 |
| OTU89 | 0.05(0.19, 0.00) | 0.06(0.50, 0.02) | 0.25 | shared | Firmicutes | Ruminococcaceae | Anaeromassilibacillus |
| OTU90 | 0.00(0.00, 0.00) | 0.01(1.52, 0.00) | 0.05 | C.unique | Firmicutes | Lachnospiraceae | Roseburia |
| OTU91 | 0.16(0.26, 0.15) | 0.05(0.12, 0.01) | < 0.01 | shared | Firmicutes | Ruminococcaceae | Ruminococcaceae UCG-002 |
| OTU92 | 0.03(0.14, 0.00) | 0.26(1.24, 0.08) | 0.02 | shared | Actinobacteria | Coriobacteriaceae | Senegalimassilia |
| OTU93 | 0.00(1.22, 0.00) | 0.00(0.00, 0.00) | 0.52 | H.unique | Firmicutes | Ruminococcaceae | Ruminococcaceae UCG-010 |
| OTU94 | 0.00(0.10, 0.00) | 0.06(0.76, 0.00) | 0.17 | shared | Firmicutes | Ruminococcaceae | Ruminococcus 2 |
| OTU95 | 0.35(1.66, 0.00) | 0.00(0.29, 0.00) | 0.12 | shared | Bacteroidetes | Bacteroidales S24-7 group | Muribaculum |
| OTU96 | 0.00(0.01, 0.00) | 0.20(0.65, 0.01) | < 0.01 | shared | Actinobacteria | Coriobacteriaceae | Enterorhabdus |
| OTU97 | 0.00(0.99, 0.00) | 0.00(0.01, 0.00) | 0.64 | shared | Firmicutes | Ruminococcaceae | Ruminococcaceae UCG-014 |
| OTU99 | 0.03(0.32, 0.02) | 0.06(0.08, 0.00) | 0.46 | shared | Firmicutes | Ruminococcaceae | Ruminiclostridium 6 |
| OTU100 | 0.05(0.30, 0.00) | 0.44(2.53, 0.09) | 0.03 | shared | Actinobacteria | Coriobacteriaceae | Olsenella |
| OTU101 | 0.31(0.89, 0.09) | 0.00(0.02, 0.00) | < 0.01 | shared | Firmicutes | Ruminococcaceae | Ruminococcaceae UCG-013 |
| OTU102 | 0.22(1.18, 0.00) | 0.08(0.17, 0.01) | 0.35 | shared | Firmicutes | Christensenellaceae | Christensenellaceae R-7 group |
| OTU103 | 0.03(0.16, 0.00) | 0.00(0.79, 0.00) | 0.39 | shared | Spirochaetae | Spirochaetaceae | Treponema 2 |
| OTU104 | 0.00(0.02, 0.00) | 0.12(0.40, 0.00) | 0.05 | shared | Firmicutes | Family XIII | [Eubacterium] nodatum group |
| OTU105 | 0.01(0.16, 0.00) | 0.37(0.76, 0.14) | 0.02 | shared | Firmicutes | Lachnospiraceae | [Ruminococcus] gauvreauii group |
| OTU106 | 0.01(0.84, 0.00) | 0.00(0.00, 0.00) | 0.13 | H.unique | Firmicutes | Lachnospiraceae | Lachnospiraceae FCS020 group |
| OTU107 | 0.02(0.05, 0.00) | 0.47(0.90, 0.03) | 0.03 | shared | Cyanobacteria | Gloeobacteraceae | Gloeobacter |
| OTU108 | 0.00(1.06, 0.00) | 0.00(0.00, 0.00) | 0.14 | H.unique | Bacteroidetes | Prevotellaceae | Prevotella 1 |
| OTU109 | 0.24(1.22, 0.00) | 0.11(0.49, 0.00) | 0.35 | shared | Bacteroidetes | Prevotellaceae | Prevotellaceae UCG-004 |
| OTU110 | 0.00(0.04, 0.00) | 0.03(1.33, 0.00) | 0.09 | shared | Firmicutes | Ruminococcaceae | Ruminococcaceae UCG-014 |
| OTU111 | 0.01(0.10, 0.00) | 0.12(0.67, 0.06) | 0.03 | shared | Firmicutes | Christensenellaceae | Christensenellaceae R-7 group |
| OTU113 | 0.06(0.42, 0.00) | 0.34(1.00, 0.12) | 0.17 | shared | Firmicutes | Lachnospiraceae | Lachnospiraceae NK3A20 group |
| OTU114 | 0.00(0.00, 0.00) | 0.00(0.94, 0.00) | 0.14 | C.unique | Firmicutes | Ruminococcaceae | Ruminococcaceae UCG-014 |
| OTU115 | 0.00(0.07, 0.00) | 0.24(0.61, 0.08) | < 0.01 | shared | Firmicutes | Christensenellaceae | Christensenellaceae R-7 group |
| OTU116 | 0.00(0.01, 0.00) | 0.39(0.84, 0.24) | < 0.01 | shared | Actinobacteria | Coriobacteriaceae | Coriobacteriaceae UCG-002 |
| OTU117 | 0.03(0.10, 0.00) | 0.08(0.41, 0.00) | 0.46 | shared | Firmicutes | Ruminococcaceae | Ruminococcaceae UCG-010 |
| OTU118 | 0.07(0.64, 0.00) | 0.04(0.30, 0.00) | 0.6 | shared | Firmicutes | Ruminococcaceae | Ruminococcaceae UCG-014 |
| OTU119 | 0.13(0.57, 0.05) | 0.00(0.01, 0.00) | < 0.01 | shared | Spirochaetae | Spirochaetaceae | Treponema 2 |
| OTU120 | 0.00(0.00, 0.00) | 0.02(0.24, 0.00) | 0.02 | C.unique | Actinobacteria | Kineococcus | Kineococcus |
| OTU121 | 0.20(0.90, 0.07) | 0.00(0.00, 0.00) | < 0.01 | H.unique | Firmicutes | Ruminococcaceae | Ruminococcaceae UCG-013 |
| OTU122 | 0.00(0.80, 0.00) | 0.00(0.00, 0.00) | 0.05 | H.unique | Firmicutes | Ruminococcaceae | Ruminococcaceae UCG-010 |
| OTU123 | 0.00(0.75, 0.00) | 0.05(0.70, 0.01) | 0.46 | shared | Spirochaetae | Spirochaetaceae | Treponema 2 |
| OTU124 | 0.03(1.91, 0.00) | 0.00(0.00, 0.00) | 0.02 | H.unique | Firmicutes | Ruminococcaceae | Ruminococcaceae UCG-014 |
| OTU126 | 0.00(0.00, 0.00) | 0.03(0.46, 0.03) | < 0.01 | C.unique | Actinobacteria | Coriobacteriaceae | Gordonibacter |
| OTU127 | 0.32(2.59, 0.04) | 0.00(0.10, 0.00) | 0.03 | shared | Firmicutes | Ruminococcaceae | Ruminococcaceae UCG-010 |
| OTU128 | 0.04(0.14, 0.01) | 0.01(0.56, 0.00) | 0.6 | shared | Firmicutes | Ruminococcaceae | Ruminococcaceae UCG-010 |
| OTU129 | 0.00(1.49, 0.00) | 0.00(0.01, 0.00) | 0.19 | shared | Bacteroidetes | Rikenellaceae | dgA-11 gut group |
| OTU130 | 0.09(0.51, 0.00) | 0.02(0.09, 0.00) | 0.4 | shared | Bacteroidetes | Rikenellaceae | Alistipes |
| OTU131 | 0.03(0.24, 0.00) | 0.23(1.49, 0.07) | 0.08 | shared | Firmicutes | Christensenellaceae | Christensenellaceae R-7 group |
| OTU133 | 0.00(0.00, 0.00) | 0.01(0.52, 0.00) | 0.05 | C.unique | Firmicutes | Ruminococcaceae | Ruminococcus 2 |
| OTU134 | 0.00(0.00, 0.00) | 0.00(0.98, 0.00) | 0.91 | C.unique | Firmicutes | Ruminococcaceae | Ruminococcus 2 |
| OTU135 | 0.06(0.58, 0.01) | 0.00(0.00, 0.00) | < 0.01 | H.unique | Firmicutes | Ruminococcaceae | Ruminococcaceae UCG-013 |
| OTU136 | 0.07(0.97, 0.05) | 0.00(0.01, 0.00) | < 0.01 | shared | Firmicutes | Ruminococcaceae | [Eubacterium] coprostanoligenes group |
| OTU137 | 0.00(0.00, 0.00) | 0.00(1.21, 0.00) | 0.14 | C.unique | Actinobacteria | Kineococcus | Kineococcus |
| OTU138 | 0.31(0.66, 0.02) | 0.09(0.22, 0.00) | 0.35 | shared | Firmicutes | Ruminococcaceae | [Eubacterium] coprostanoligenes group |
| OTU139 | 0.07(0.53, 0.03) | 0.00(0.06, 0.00) | 0.03 | shared | Firmicutes | Acidaminococcaceae | Phascolarctobacterium |
| OTU140 | 0.26(0.73, 0.08) | 0.01(0.01, 0.00) | < 0.01 | shared | Firmicutes | Ruminococcaceae | Ruminococcaceae UCG-013 |
| OTU141 | 0.15(0.35, 0.02) | 0.00(0.01, 0.00) | < 0.01 | shared | Firmicutes | Ruminococcaceae | Ruminococcaceae UCG-010 |
| OTU142 | 0.00(0.00, 0.00) | 0.02(0.37, 0.00) | 0.22 | C.unique | Firmicutes | Lachnospiraceae | [Eubacterium] ventriosum group |
| OTU143 | 0.00(0.42, 0.00) | 0.02(0.12, 0.00) | 0.28 | shared | Firmicutes | Ruminococcaceae | Ruminococcaceae UCG-014 |
| OTU144 | 0.37(0.59, 0.18) | 0.41(0.54, 0.07) | 0.75 | shared | Firmicutes | Christensenellaceae | Christensenellaceae R-7 group |
| OTU145 | 0.21(0.41, 0.03) | 0.00(0.01, 0.00) | < 0.01 | shared | Firmicutes | Ruminococcaceae | Ruminococcaceae UCG-013 |
| OTU146 | 0.00(0.01, 0.00) | 0.01(1.15, 0.00) | 0.02 | shared | Firmicutes | Ruminococcaceae | Anaerotruncus |
| OTU147 | 0.00(0.00, 0.00) | 0.00(0.86, 0.00) | 0.14 | C.unique | Spirochaetae | Spirochaetaceae | Treponema 2 |
| OTU148 | 0.05(0.39, 0.00) | 0.03(0.17, 0.01) | 0.92 | shared | Firmicutes | Lachnospiraceae | Pseudobutyrivibrio |
| OTU149 | 0.03(0.79, 0.00) | 0.00(0.02, 0.00) | 0.33 | shared | Firmicutes | Ruminococcaceae | Ruminococcaceae UCG-014 |
| OTU150 | 0.01(0.02, 0.00) | 0.08(0.41, 0.03) | < 0.01 | shared | Actinobacteria | Coriobacteriaceae | Adlercreutzia |
| OTU151 | 0.10(0.13, 0.01) | 0.00(0.03, 0.00) | 0.03 | shared | Firmicutes | Family XIII | Family XIII UCG-002 |
| OTU152 | 0.00(0.00, 0.00) | 0.01(0.84, 0.00) | 0.02 | C.unique | Bacteroidetes | Paludibacteraceae | Paludibacter |
| OTU153 | 0.05(0.18, 0.02) | 0.01(0.04, 0.00) | 0.07 | shared | Firmicutes | Ruminococcaceae | Ruminococcaceae UCG-005 |
| OTU154 | 0.12(0.72, 0.05) | 0.00(0.07, 0.00) | 0.02 | shared | Firmicutes | Ruminococcaceae | Ruminococcaceae UCG-007 |
| OTU155 | 0.00(0.00, 0.00) | 0.30(0.65, 0.00) | 0.03 | C.unique | Saccharibacteria | Unknown Family | Candidatus Saccharimonas |
| OTU157 | 0.03(0.27, 0.00) | 0.00(0.04, 0.00) | 0.45 | shared | Bacteroidetes | Bacteroidales BS11 gut group | Phaeocystidibacter luteus |
| OTU158 | 0.03(0.04, 0.00) | 0.06(0.49, 0.01) | 0.35 | shared | Firmicutes | Lachnospiraceae | Acetitomaculum |
| OTU159 | 0.02(0.09, 0.00) | 0.08(0.29, 0.04) | 0.08 | shared | Firmicutes | Erysipelotrichaceae | Erysipelotrichaceae UCG-008 |
| OTU160 | 0.06(0.37, 0.02) | 0.00(0.00, 0.00) | < 0.01 | shared | Bacteroidetes | Rikenellaceae | Alistipes |
| OTU161 | 0.00(0.00, 0.00) | 0.20(0.21, 0.00) | < 0.01 | shared | Firmicutes | Family XIII | Family XIII AD3011 group |
| OTU163 | 0.01(0.32, 0.00) | 0.00(0.00, 0.00) | 0.02 | H.unique | Firmicutes | Ruminococcaceae | Ruminococcus 1 |
| OTU164 | 0.03(0.48, 0.01) | 0.01(0.01, 0.00) | 0.05 | shared | Firmicutes | Ruminococcaceae | Oscillibacter |
| OTU165 | 0.00(0.67, 0.00) | 0.00(0.11, 0.00) | 0.41 | shared | Bacteroidetes | Bacteroidaceae | Bacteroides |
| OTU166 | 0.06(0.25, 0.00) | 0.00(0.01, 0.00) | 0.13 | shared | Firmicutes | Ruminococcaceae | Ruminococcus 1 |
| OTU167 | 0.09(0.57, 0.02) | 0.00(0.01, 0.00) | < 0.01 | shared | Firmicutes | Family XIII | Family XIII AD3011 group |
| OTU168 | 0.01(0.02, 0.00) | 0.09(0.20, 0.07) | < 0.01 | shared | Firmicutes | Christensenellaceae | Christensenellaceae R-7 group |
| OTU169 | 0.00(0.00, 0.00) | 0.08(0.27, 0.00) | 0.02 | C.unique | Actinobacteria | Coriobacteriaceae | Senegalimassilia |
| OTU170 | 0.00(0.80, 0.00) | 0.00(0.00, 0.00) | 0.37 | H.unique | Bacteroidetes | Porphyromonadaceae | Barnesiella |
| OTU171 | 0.11(0.50, 0.04) | 0.00(0.00, 0.00) | < 0.01 | H.unique | Firmicutes | Ruminococcaceae | [Eubacterium] coprostanoligenes group |
| OTU172 | 0.00(0.00, 0.00) | 0.01(0.65, 0.00) | 0.05 | shared | Proteobacteria | Succinivibrionaceae | Succinivibrio |
| OTU173 | 0.02(0.32, 0.00) | 0.00(0.01, 0.00) | 0.05 | shared | Firmicutes | Ruminococcaceae | Ruminococcaceae UCG-010 |
| OTU174 | 0.05(0.47, 0.01) | 0.03(0.08, 0.00) | 0.75 | shared | Firmicutes | Christensenellaceae | Christensenellaceae R-7 group |
| OTU175 | 0.01(0.01, 0.00) | 0.12(0.20, 0.00) | 0.09 | shared | Firmicutes | Lachnospiraceae | Acetitomaculum |
| OTU176 | 0.01(0.03, 0.00) | 0.01(0.38, 0.00) | 0.68 | shared | Actinobacteria | Coriobacteriaceae | Olsenella |
| OTU177 | 0.09(0.16, 0.05) | 0.04(0.10, 0.00) | 0.08 | shared | Firmicutes | Ruminococcaceae | Ruminococcaceae UCG-009 |
| OTU179 | 0.02(0.18, 0.00) | 0.00(0.01, 0.00) | 0.13 | shared | Firmicutes | Clostridiales vadinBB60 group | Lutispora |
| OTU180 | 0.12(0.41, 0.03) | 0.00(0.01, 0.00) | < 0.01 | shared | Firmicutes | Ruminococcaceae | Ruminococcaceae UCG-013 |
| OTU181 | 0.02(0.04, 0.01) | 0.03(0.21, 0.01) | 0.75 | shared | Firmicutes | Lachnospiraceae | Lachnoclostridium |
| OTU182 | 1.02(3.94, 0.90) | 0.04(0.06, 0.01) | < 0.01 | shared | Firmicutes | Christensenellaceae | Christensenellaceae R-7 group |
| OTU183 | 0.07(0.13, 0.01) | 0.02(0.12, 0.00) | 0.25 | shared | Firmicutes | Erysipelotrichaceae | Erysipelothrix |
| OTU184 | 0.00(0.27, 0.00) | 0.00(0.06, 0.00) | 0.52 | shared | Firmicutes | Ruminococcaceae | Ruminococcaceae UCG-014 |
| OTU185 | 0.05(0.30, 0.00) | 0.00(0.02, 0.00) | 0.14 | shared | Firmicutes | Lachnospiraceae | [Ruminococcus] gnavus |
| OTU186 | 0.00(0.44, 0.00) | 0.29(0.38, 0.10) | 0.12 | shared | Firmicutes | Christensenellaceae | Christensenellaceae R-7 group |
| OTU187 | 0.07(0.19, 0.07) | 0.00(0.05, 0.00) | < 0.01 | shared | Firmicutes | Ruminococcaceae | Ruminiclostridium 6 |
| OTU188 | 0.02(0.07, 0.00) | 0.30(0.36, 0.17) | < 0.01 | shared | Firmicutes | Lachnospiraceae | Lachnospiraceae UCG-002 |
| OTU191 | 0.02(0.04, 0.00) | 0.07(0.26, 0.03) | 0.03 | shared | Firmicutes | Lachnospiraceae | Syntrophococcus |
| OTU193 | 0.05(0.06, 0.01) | 0.19(0.58, 0.11) | < 0.01 | shared | Firmicutes | Family XIII | Family XIII AD3011 group |
| OTU194 | 0.00(0.28, 0.00) | 0.01(0.08, 0.00) | 0.83 | shared | Bacteroidetes | Bacteroidales S24-7 group | Muribaculum |
| OTU196 | 0.05(0.26, 0.04) | 0.01(0.05, 0.00) | 0.08 | shared | Firmicutes | Ruminococcaceae | Ruminococcaceae UCG-005 |
| OTU197 | 0.01(0.07, 0.00) | 0.14(0.27, 0.06) | 0.03 | shared | Firmicutes | Eubacteriaceae | Anaerofustis |
| OTU198 | 0.00(0.00, 0.00) | 0.29(0.48, 0.00) | 0.02 | C.unique | Firmicutes | Planococcaceae | Rummeliibacillus |
| OTU199 | 0.03(0.07, 0.00) | 0.06(1.06, 0.01) | 0.17 | shared | Firmicutes | Lachnospiraceae | Blautia |
| OTU200 | 0.01(0.03, 0.00) | 0.01(0.35, 0.01) | 0.46 | shared | Firmicutes | Lachnospiraceae | Acetitomaculum |
| OTU203 | 0.10(0.28, 0.00) | 0.00(0.00, 0.00) | < 0.01 | H.unique | Firmicutes | Ruminococcaceae | [Eubacterium] coprostanoligenes group |
| OTU204 | 0.08(0.52, 0.01) | 0.00(0.01, 0.00) | 0.02 | shared | Firmicutes | Clostridiales vadinBB60 group | Christensenella |
| OTU205 | 0.01(0.48, 0.00) | 0.00(0.00, 0.00) | 0.13 | H.unique | Firmicutes | Ruminococcaceae | Ruminococcaceae UCG-010 |
| OTU206 | 0.00(0.61, 0.00) | 0.00(0.01, 0.00) | 0.52 | shared | Proteobacteria | Rhodospirillaceae | Aestuariispira |
| OTU207 | 0.02(0.16, 0.00) | 0.01(0.04, 0.00) | 0.17 | shared | Firmicutes | Ruminococcaceae | [Eubacterium] coprostanoligenes group |
| OTU208 | 0.18(0.66, 0.12) | 0.00(0.01, 0.00) | < 0.01 | shared | Firmicutes | Lachnospiraceae | Lachnospiraceae NK4A136 group |
| OTU209 | 0.07(0.13, 0.04) | 0.00(0.00, 0.00) | < 0.01 | H.unique | Bacteroidetes | Prevotellaceae | Prevotellaceae UCG-001 |
| OTU211 | 0.00(0.00, 0.00) | 0.06(0.31, 0.00) | 0.13 | C.unique | Firmicutes | Lachnospiraceae | Lachnospiraceae XPB1014 group |
| OTU212 | 0.00(0.00, 0.00) | 0.00(0.23, 0.00) | 0.37 | C.unique | Firmicutes | Erysipelotrichaceae | Solobacterium |
| OTU213 | 0.00(0.14, 0.00) | 0.07(0.51, 0.02) | 0.17 | shared | Actinobacteria | Coriobacteriaceae | Senegalimassilia |
| OTU214 | 0.00(0.00, 0.00) | 0.01(0.45, 0.00) | 0.03 | C.unique | Firmicutes | Lachnospiraceae | Marvinbryantia |
| OTU216 | 0.00(0.78, 0.00) | 0.00(0.07, 0.00) | 0.91 | shared | Bacteroidetes | Bacteroidaceae | Bacteroides |
| OTU217 | 0.00(0.00, 0.00) | 0.04(0.24, 0.00) | 0.02 | C.unique | Firmicutes | Eubacteriaceae | Eubacterium |
| OTU218 | 0.00(0.00, 0.00) | 0.03(0.41, 0.01) | < 0.01 | C.unique | Actinobacteria | Coriobacteriaceae | Slackia |
| OTU219 | 0.09(0.29, 0.06) | 0.03(0.07, 0.00) | 0.02 | shared | Firmicutes | Ruminococcaceae | Ruminococcaceae NK4A214 group |
| OTU220 | 0.02(0.30, 0.00) | 0.00(0.00, 0.00) | 0.02 | H.unique | Firmicutes | Ruminococcaceae | Ruminococcaceae UCG-013 |
| OTU222 | 0.01(0.16, 0.00) | 0.48(0.62, 0.23) | < 0.01 | shared | Firmicutes | Family XIII | Family XIII AD3011 group |
| OTU223 | 0.02(0.15, 0.01) | 0.00(0.02, 0.00) | 0.04 | shared | Firmicutes | Lachnospiraceae | [Clostridium] saccharolyticum |
| OTU224 | 0.01(0.07, 0.00) | 0.05(0.13, 0.02) | 0.05 | shared | Actinobacteria | Fodinicola | Fodinicola |
| OTU225 | 0.01(0.11, 0.00) | 0.00(0.00, 0.00) | 0.05 | H.unique | Bacteroidetes | Bacteroidales UCG-001 | Bacteroides |
| OTU226 | 0.26(0.39, 0.02) | 0.02(0.05, 0.00) | 0.05 | shared | Firmicutes | Peptococcaceae | Desulfosporosinus |
| OTU227 | 0.01(0.04, 0.00) | 0.04(0.19, 0.00) | 0.25 | shared | Firmicutes | Ruminococcaceae | Anaerotruncus |
| OTU228 | 0.05(0.23, 0.00) | 0.00(0.02, 0.00) | 0.08 | shared | Bacteroidetes | Rikenellaceae | Rikenellaceae RC9 gut group |
| OTU229 | 0.04(0.28, 0.00) | 0.00(0.02, 0.00) | 0.07 | shared | Bacteroidetes | Bacteroidaceae | Bacteroides |
| OTU230 | 0.00(0.01, 0.00) | 0.05(0.28, 0.00) | 0.06 | shared | Bacteroidetes | Rikenellaceae | Rikenellaceae RC9 gut group |
| OTU232 | 0.14(0.60, 0.09) | 0.01(0.05, 0.00) | < 0.01 | shared | Firmicutes | Ruminococcaceae | Oscillibacter |
| OTU233 | 0.00(0.27, 0.00) | 0.00(0.00, 0.00) | 0.14 | H.unique | Firmicutes | Ruminococcaceae | Anaerotruncus |
| OTU234 | 0.00(0.00, 0.00) | 0.85(1.53, 0.15) | < 0.01 | C.unique | Saccharibacteria | Unknown Family | Candidatus Saccharimonas |
| OTU235 | 0.10(0.28, 0.00) | 0.00(0.03, 0.00) | 0.04 | shared | Firmicutes | Ruminococcaceae | Ruminococcaceae UCG-013 |
| OTU236 | 0.06(0.59, 0.02) | 0.00(0.00, 0.00) | < 0.01 | H.unique | Firmicutes | Lachnospiraceae | [Clostridium] propionicum |
| OTU237 | 0.00(0.03, 0.00) | 0.16(0.24, 0.09) | < 0.01 | shared | Firmicutes | Lachnospiraceae | Acetitomaculum |
| OTU238 | 0.01(0.01, 0.00) | 0.25(0.34, 0.04) | < 0.01 | shared | Actinobacteria | Coriobacteriaceae | Senegalimassilia |
| OTU239 | 0.06(0.28, 0.01) | 0.00(0.02, 0.00) | 0.07 | shared | Firmicutes | Ruminococcaceae | Ruminococcaceae UCG-013 |
| OTU240 | 0.03(0.27, 0.00) | 0.00(0.10, 0.00) | 0.28 | shared | Firmicutes | Christensenellaceae | Christensenellaceae R-7 group |
| OTU241 | 0.01(0.11, 0.00) | 0.02(0.08, 0.00) | 0.83 | shared | Firmicutes | Ruminococcaceae | Anaerotruncus |
| OTU243 | 0.00(0.02, 0.00) | 0.06(0.11, 0.04) | < 0.01 | shared | Actinobacteria | Micrococcaceae | Nesterenkonia |
| OTU245 | 0.00(0.00, 0.00) | 0.02(0.19, 0.00) | 0.05 | C.unique | Bacteroidetes | Rikenellaceae | Alistipes |
| OTU246 | 0.00(0.02, 0.00) | 0.11(0.29, 0.06) | < 0.01 | shared | Firmicutes | Lachnospiraceae | [Ruminococcus] gnavus |
| OTU247 | 0.11(0.29, 0.00) | 0.02(0.03, 0.00) | 0.24 | shared | Firmicutes | Ruminococcaceae | Anaerofilum |
| OTU248 | 0.06(0.12, 0.00) | 0.02(0.03, 0.00) | 0.29 | shared | Firmicutes | Ruminococcaceae | Caproiciproducens |
| OTU249 | 0.02(0.10, 0.00) | 0.08(0.40, 0.00) | 0.12 | shared | Bacteroidetes | Rikenellaceae | dgA-11 gut group |
| OTU250 | 0.00(0.04, 0.00) | 0.02(0.40, 0.00) | 0.28 | shared | Firmicutes | Ruminococcaceae | Ruminococcus 2 |
| OTU251 | 0.16(0.27, 0.00) | 0.02(0.03, 0.00) | 0.4 | shared | Firmicutes | Ruminococcaceae | Pseudoflavonifractor |
| OTU252 | 0.00(0.00, 0.00) | 0.00(0.41, 0.00) | 0.19 | C.unique | Firmicutes | Ruminococcaceae | Ruminococcaceae UCG-014 |
| OTU253 | 0.06(0.41, 0.00) | 0.00(0.02, 0.00) | 0.07 | shared | Bacteroidetes | Prevotellaceae | Prevotellaceae UCG-004 |
| OTU254 | 0.02(0.13, 0.00) | 0.00(0.00, 0.00) | 0.13 | H.unique | Bacteroidetes | Prevotellaceae | Prevotellaceae UCG-001 |
| OTU255 | 0.00(0.00, 0.00) | 0.05(0.40, 0.00) | 0.05 | C.unique | Firmicutes | Ruminococcaceae | Ruminococcaceae UCG-014 |
| OTU257 | 0.03(0.24, 0.00) | 0.00(0.04, 0.00) | 0.32 | shared | Firmicutes | Ruminococcaceae | [Eubacterium] coprostanoligenes group |
| OTU258 | 0.01(0.05, 0.00) | 0.35(0.62, 0.17) | < 0.01 | shared | Firmicutes | Family XIII | Mogibacterium |
| OTU261 | 0.00(0.01, 0.00) | 0.01(0.25, 0.00) | 0.06 | shared | Firmicutes | Lachnospiraceae | [Eubacterium] hallii group |
| OTU262 | 0.00(0.01, 0.00) | 0.16(0.46, 0.04) | < 0.01 | shared | Firmicutes | Caldicoprobacteraceae | Caldicoprobacter |
| OTU264 | 0.00(0.16, 0.00) | 0.01(0.02, 0.00) | 0.83 | shared | Spirochaetae | Spirochaetaceae | Treponema 2 |
| OTU265 | 0.00(0.00, 0.00) | 0.04(0.15, 0.00) | < 0.01 | C.unique | Actinobacteria | Coriobacteriaceae | Denitrobacterium |
| OTU266 | 0.00(0.61, 0.00) | 0.00(0.00, 0.00) | 0.14 | H.unique | Bacteroidetes | Prevotellaceae | Prevotella 1 |
| OTU267 | 0.00(0.02, 0.00) | 0.10(0.16, 0.00) | 0.07 | shared | Actinobacteria | Streptomyces | Streptomyces |
| OTU268 | 0.04(0.15, 0.00) | 0.00(0.00, 0.00) | 0.02 | H.unique | Firmicutes | Lachnospiraceae | [Eubacterium] ruminantium group |
| OTU269 | 0.07(0.14, 0.00) | 0.00(0.00, 0.00) | 0.02 | H.unique | Bacteroidetes | Bacteroidales S24-7 group | Barnesiella |
| OTU271 | 0.00(0.00, 0.00) | 0.31(0.41, 0.03) | < 0.01 | C.unique | Saccharibacteria | Unknown Family | Candidatus Saccharimonas |
| OTU273 | 0.07(0.19, 0.01) | 0.01(0.17, 0.01) | 0.92 | shared | Firmicutes | Christensenellaceae | Christensenellaceae R-7 group |
| OTU275 | 0.00(0.71, 0.00) | 0.00(0.00, 0.00) | 0.14 | H.unique | Bacteroidetes | Bacteroidales S24-7 group | Barnesiella |
| OTU276 | 0.06(0.21, 0.02) | 0.03(0.13, 0.00) | 0.12 | shared | Firmicutes | Ruminococcaceae | Pseudoflavonifractor |
| OTU278 | 0.05(0.24, 0.01) | 0.01(0.03, 0.00) | 0.03 | shared | Bacteroidetes | Rikenellaceae | Alistipes |
| OTU279 | 0.00(0.01, 0.00) | 0.01(0.32, 0.00) | 0.13 | shared | Actinobacteria | Kineococcus | Kineosporia |
| OTU280 | 0.01(0.25, 0.00) | 0.00(0.00, 0.00) | 0.05 | H.unique | Bacteroidetes | Rikenellaceae | Rikenellaceae RC9 gut group |
| OTU281 | 0.00(0.00, 0.00) | 0.05(0.96, 0.02) | < 0.01 | shared | Actinobacteria | Coriobacteriaceae | Senegalimassilia |
| OTU282 | 0.00(0.72, 0.00) | 0.00(0.00, 0.00) | 0.37 | H.unique | Bacteroidetes | Prevotellaceae | Prevotellaceae UCG-003 |
| OTU283 | 0.00(0.01, 0.00) | 0.02(0.35, 0.00) | 0.07 | shared | Firmicutes | Christensenellaceae | Christensenellaceae R-7 group |
| OTU286 | 0.01(0.10, 0.00) | 0.07(0.12, 0.01) | 0.12 | shared | Actinobacteria | Coriobacteriaceae | Coriobacterium |
| OTU287 | 0.00(0.11, 0.00) | 0.00(0.01, 0.00) | 0.29 | shared | Firmicutes | Lachnospiraceae | Tyzzerella |
| OTU288 | 0.00(0.12, 0.00) | 0.03(0.11, 0.00) | 0.28 | shared | Firmicutes | Ruminococcaceae | Ruminococcaceae UCG-014 |
| OTU289 | 0.00(0.00, 0.00) | 0.10(0.37, 0.03) | < 0.01 | C.unique | Actinobacteria | Modestobacter | Modestobacter roseus |
| OTU290 | 0.00(0.04, 0.00) | 0.05(0.25, 0.00) | 0.05 | shared | Firmicutes | Lachnospiraceae | Syntrophococcus |
| OTU291 | 0.01(0.24, 0.00) | 0.01(0.04, 0.00) | 1 | shared | Bacteroidetes | Bacteroidales Incertae Sedis | Phocaeicola |
| OTU292 | 0.00(0.02, 0.00) | 0.11(0.22, 0.02) | 0.02 | shared | Firmicutes | Lachnospiraceae | Howardella |
| OTU293 | 0.03(0.21, 0.00) | 0.00(0.03, 0.00) | 0.17 | shared | Bacteroidetes | Rikenellaceae | Alistipes |
| OTU295 | 0.00(0.00, 0.00) | 0.02(0.56, 0.00) | 0.05 | C.unique | Firmicutes | Clostridiaceae 1 | Clostridium sensu stricto 1 |
| OTU297 | 0.00(0.01, 0.00) | 0.10(0.21, 0.01) | 0.02 | shared | Firmicutes | Lachnospiraceae | Roseburia |
| OTU298 | 0.12(0.22, 0.07) | 0.00(0.01, 0.00) | < 0.01 | shared | Firmicutes | Ruminococcaceae | Ruminococcaceae UCG-010 |
| OTU301 | 0.00(0.00, 0.00) | 0.02(0.28, 0.00) | 0.02 | C.unique | Firmicutes | Ruminococcaceae | Ruminococcaceae UCG-014 |
| OTU302 | 0.24(1.03, 0.00) | 0.04(0.32, 0.00) | 0.17 | shared | Firmicutes | Ruminococcaceae | Ruminococcaceae UCG-014 |
| OTU305 | 0.04(0.19, 0.00) | 0.01(0.01, 0.00) | 0.33 | shared | Firmicutes | Ruminococcaceae | Ruminiclostridium 5 |
| OTU306 | 0.04(0.17, 0.00) | 0.00(0.01, 0.00) | 0.01 | shared | Firmicutes | Lachnospiraceae | Roseburia |
| OTU307 | 0.04(0.12, 0.00) | 0.00(0.02, 0.00) | 0.13 | shared | Tenericutes | Acholeplasmataceae | Acholeplasma |
| OTU308 | 0.02(0.22, 0.00) | 0.00(0.02, 0.00) | 0.35 | shared | Firmicutes | Christensenellaceae | Christensenellaceae R-7 group |
| OTU310 | 0.33(0.70, 0.07) | 0.06(0.11, 0.00) | 0.03 | shared | Firmicutes | Christensenellaceae | Christensenellaceae R-7 group |
| OTU312 | 0.00(0.00, 0.00) | 0.00(0.33, 0.00) | 0.14 | C.unique | Actinobacteria | Modestobacter | Modestobacter muralis |
| OTU314 | 0.01(0.09, 0.00) | 0.01(0.15, 0.00) | 0.83 | shared | Tenericutes | Acholeplasmataceae | Acholeplasma |
| OTU316 | 0.00(0.01, 0.00) | 0.01(0.32, 0.00) | 0.12 | shared | Actinobacteria | Coriobacteriaceae | Gordonibacter |
| OTU321 | 0.00(0.00, 0.00) | 0.01(0.22, 0.01) | < 0.01 | C.unique | Actinobacteria | Coriobacteriaceae | Paraeggerthella |
| OTU323 | 0.02(0.16, 0.00) | 0.00(0.00, 0.00) | < 0.01 | shared | Bacteroidetes | Bacteroidales Incertae Sedis | Phocaeicola |
| OTU326 | 0.00(0.20, 0.00) | 0.11(0.46, 0.03) | 0.07 | shared | Actinobacteria | Kineococcus | Kineococcus |
| OTU327 | 0.01(0.19, 0.00) | 0.00(0.04, 0.00) | 0.46 | shared | Firmicutes | Ruminococcaceae | Ruminococcaceae UCG-014 |
| OTU328 | 0.03(0.10, 0.00) | 0.00(0.00, 0.00) | < 0.01 | H.unique | Bacteroidetes | Porphyromonadaceae | Butyricimonas |
| OTU329 | 0.04(0.20, 0.00) | 0.00(0.00, 0.00) | 0.02 | H.unique | Firmicutes | Ruminococcaceae | Ruminococcaceae UCG-010 |
| OTU331 | 0.08(0.16, 0.01) | 0.00(0.02, 0.00) | 0.02 | shared | Bacteroidetes | Rikenellaceae | Alistipes |
| OTU333 | 0.00(0.00, 0.00) | 0.00(0.35, 0.00) | 0.88 | C.unique | Firmicutes | Ruminococcaceae | Ruminococcaceae UCG-010 |
| OTU334 | 0.00(0.07, 0.00) | 0.02(0.11, 0.00) | 0.52 | shared | Firmicutes | Ruminococcaceae | Ruminococcaceae UCG-010 |
| OTU336 | 0.00(0.16, 0.00) | 0.11(0.26, 0.00) | 0.19 | shared | Bacteroidetes | Bacteroidaceae | Bacteroides |
| OTU337 | 0.02(0.15, 0.00) | 0.00(0.01, 0.00) | 0.13 | shared | Firmicutes | Ruminococcaceae | Caproiciproducens |
| OTU338 | 0.00(0.01, 0.00) | 0.09(0.19, 0.01) | < 0.01 | shared | Actinobacteria | Nonomuraea | Nonomuraea |
| OTU339 | 0.00(0.08, 0.00) | 0.04(0.24, 0.00) | 0.13 | shared | Proteobacteria | Rhodospirillaceae | Aestuariispira |
| OTU340 | 0.00(0.22, 0.00) | 0.08(0.33, 0.01) | 0.07 | shared | Firmicutes | Ruminococcaceae | Ruminococcus 2 |
| OTU341 | 0.01(0.16, 0.00) | 0.00(0.01, 0.00) | 0.13 | shared | Firmicutes | Lachnospiraceae | Lachnoclostridium 10 |
| OTU343 | 0.00(0.02, 0.00) | 0.00(0.10, 0.00) | 0.7 | shared | Firmicutes | Veillonellaceae | Schwartzia |
| OTU344 | 0.01(0.19, 0.00) | 0.00(0.02, 0.00) | 0.29 | shared | Firmicutes | Christensenellaceae | Christensenellaceae R-7 group |
| OTU345 | 0.00(0.00, 0.00) | 0.01(0.23, 0.00) | 0.03 | C.unique | Actinobacteria | Kineococcus | Kineococcus |
| OTU346 | 0.01(0.24, 0.00) | 0.00(0.00, 0.00) | < 0.01 | H.unique | Bacteroidetes | Rikenellaceae | Rikenellaceae RC9 gut group |
| OTU349 | 0.06(0.26, 0.00) | 0.00(0.00, 0.00) | 0.03 | H.unique | Firmicutes | Ruminococcaceae | Ruminococcaceae UCG-010 |
| OTU351 | 0.01(0.01, 0.00) | 0.03(0.17, 0.00) | 0.12 | shared | Firmicutes | Lachnospiraceae | Acetitomaculum |
| OTU354 | 0.00(0.00, 0.00) | 0.11(0.22, 0.00) | 0.02 | shared | Firmicutes | Ruminococcaceae | Ruminococcaceae UCG-014 |
| OTU355 | 0.00(0.00, 0.00) | 0.02(0.34, 0.00) | < 0.01 | shared | Firmicutes | Caldicoprobacteraceae | Caldicoprobacter |
| OTU359 | 0.04(0.18, 0.00) | 0.00(0.01, 0.00) | 0.01 | shared | Firmicutes | Ruminococcaceae | Ruminococcus 1 |
| OTU360 | 0.04(0.18, 0.00) | 0.00(0.02, 0.00) | 0.07 | shared | Firmicutes | Clostridiales vadinBB60 group | Christensenella |
| OTU362 | 0.00(0.00, 0.00) | 0.19(0.32, 0.15) | < 0.01 | H.unique | Saccharibacteria | Unknown Family | Candidatus Saccharimonas |
| OTU363 | 0.00(0.01, 0.00) | 0.01(0.11, 0.00) | 0.2 | shared | Firmicutes | Clostridiales vadinBB60 group | Christensenella |
| OTU364 | 0.03(0.14, 0.00) | 0.00(0.00, 0.00) | 0.03 | H.unique | Firmicutes | Ruminococcaceae | Ruminococcaceae UCG-010 |
| OTU367 | 0.03(0.22, 0.01) | 0.00(0.12, 0.00) | 0.12 | shared | Firmicutes | Ruminococcaceae | Caproiciproducens |
| OTU368 | 0.00(0.11, 0.00) | 0.01(0.03, 0.00) | 0.91 | shared | Firmicutes | Ruminococcaceae | Ruminococcaceae UCG-010 |
| OTU370 | 0.00(0.16, 0.00) | 0.00(0.00, 0.00) | 0.19 | H.unique | Firmicutes | Ruminococcaceae | Ruminococcaceae UCG-013 |
| OTU371 | 0.00(0.04, 0.00) | 0.02(0.24, 0.00) | 0.15 | shared | Firmicutes | Ruminococcaceae | Ruminococcaceae UCG-010 |
| OTU372 | 0.02(0.14, 0.00) | 0.02(0.04, 0.00) | 0.46 | shared | Firmicutes | Lachnospiraceae | Acetitomaculum |
| OTU373 | 0.15(0.95, 0.00) | 0.03(0.05, 0.00) | 0.24 | shared | Firmicutes | Christensenellaceae | Christensenellaceae R-7 group |
| OTU374 | 0.01(0.11, 0.00) | 0.00(0.06, 0.00) | 0.75 | shared | Firmicutes | Ruminococcaceae | Ruminococcaceae UCG-010 |
| OTU375 | 0.00(0.53, 0.00) | 0.00(0.00, 0.00) | 0.05 | H.unique | Bacteroidetes | Porphyromonadaceae | Parabacteroides |
| OTU378 | 0.00(0.17, 0.00) | 0.00(0.00, 0.00) | 0.14 | H.unique | Bacteroidetes | Prevotellaceae | Prevotellaceae UCG-004 |
| OTU379 | 0.00(0.01, 0.00) | 0.00(0.24, 0.00) | 0.09 | shared | Firmicutes | Lachnospiraceae | Lachnospiraceae NK3A20 group |
| OTU380 | 0.09(0.34, 0.01) | 0.00(0.01, 0.00) | 0.03 | shared | Firmicutes | Ruminococcaceae | Ruminococcaceae UCG-013 |
| OTU382 | 0.00(0.01, 0.00) | 0.09(0.10, 0.02) | < 0.01 | shared | Cyanobacteria | Gloeobacteraceae | Gloeobacter |
| OTU383 | 0.00(0.01, 0.00) | 0.03(0.16, 0.00) | 0.03 | shared | Actinobacteria | Coriobacteriaceae | Slackia |
| OTU385 | 0.00(0.25, 0.00) | 0.00(0.00, 0.00) | 0.05 | H.unique | Bacteroidetes | Bacteroidales S24-7 group | Muribaculum |
| OTU386 | 0.03(0.12, 0.03) | 0.00(0.01, 0.00) | < 0.01 | shared | Firmicutes | Christensenellaceae | Christensenellaceae R-7 group |
| OTU387 | 0.05(0.17, 0.00) | 0.02(0.04, 0.00) | 0.24 | shared | Firmicutes | Ruminococcaceae | Ruminiclostridium |
| OTU388 | 0.00(0.18, 0.00) | 0.00(0.00, 0.00) | 0.14 | H.unique | Firmicutes | Ruminococcaceae | Ruminococcaceae UCG-013 |
| OTU390 | 0.02(0.21, 0.00) | 0.00(0.00, 0.00) | 0.03 | H.unique | Firmicutes | Ruminococcaceae | Ruminococcaceae UCG-013 |
| OTU391 | 0.00(0.00, 0.00) | 0.00(0.16, 0.00) | 0.14 | C.unique | Firmicutes | Lachnospiraceae | Cellulosilyticum |
| OTU393 | 0.00(0.00, 0.00) | 0.01(0.15, 0.00) | 0.05 | C.unique | Firmicutes | Erysipelotrichaceae | Holdemania |
| OTU394 | 0.00(0.01, 0.00) | 0.06(0.11, 0.01) | 0.02 | shared | Firmicutes | Lachnospiraceae | Coprococcus 1 |
| OTU395 | 0.00(0.00, 0.00) | 0.06(0.28, 0.00) | 0.05 | C.unique | Actinobacteria | Kineococcus | Kineococcus |
| OTU397 | 0.00(0.03, 0.00) | 0.02(0.25, 0.00) | 0.17 | shared | Actinobacteria | Coriobacteriaceae | Parvibacter |
| OTU400 | 0.00(0.14, 0.00) | 0.00(0.09, 0.00) | 0.22 | shared | Bacteroidetes | Prevotellaceae | Prevotellaceae UCG-004 |
| OTU401 | 0.00(0.03, 0.00) | 0.01(0.18, 0.00) | 0.09 | shared | Tenericutes | Spiroplasmataceae | Spiroplasma |
| OTU404 | 0.02(0.07, 0.01) | 0.01(0.18, 0.00) | 0.46 | shared | Firmicutes | Lachnospiraceae | [Eubacterium] rectale group |
| OTU405 | 0.02(0.15, 0.00) | 0.00(0.02, 0.00) | 0.19 | shared | Firmicutes | Christensenellaceae | Christensenellaceae R-7 group |
| OTU407 | 0.01(0.03, 0.00) | 0.04(0.11, 0.01) | 0.07 | shared | Actinobacteria | Coriobacteriaceae | Paraeggerthella |
| OTU411 | 0.00(0.02, 0.00) | 0.06(0.23, 0.01) | 0.02 | shared | Actinobacteria | Coriobacteriaceae | Atopobium |
| OTU413 | 0.00(0.00, 0.00) | 0.10(0.14, 0.00) | 0.02 | C.unique | Firmicutes | Lachnospiraceae | Acetitomaculum |
| OTU416 | 0.00(0.00, 0.00) | 0.01(0.17, 0.00) | 0.05 | C.unique | Actinobacteria | Fodinicola | Fodinicola |
| OTU417 | 0.03(0.11, 0.01) | 0.16(0.39, 0.06) | 0.02 | shared | Firmicutes | Family XIII | Family XIII AD3011 group |
| OTU418 | 0.00(0.12, 0.00) | 0.00(0.00, 0.00) | 0.14 | H.unique | Firmicutes | Ruminococcaceae | Ruminococcaceae UCG-013 |
| OTU422 | 0.00(0.22, 0.00) | 0.00(0.00, 0.00) | 0.14 | H.unique | Bacteroidetes | Bacteroidales S24-7 group | Muribaculum |
| OTU423 | 0.00(0.00, 0.00) | 0.01(0.16, 0.00) | 0.05 | C.unique | Firmicutes | Ruminococcaceae | Ruminococcaceae UCG-001 |
| OTU426 | 0.01(0.12, 0.00) | 0.00(0.01, 0.00) | 0.13 | shared | Firmicutes | Ruminococcaceae | Ruminococcus 1 |
| OTU427 | 0.00(0.02, 0.00) | 0.05(0.15, 0.01) | 0.02 | shared | Firmicutes | Family XIII | [Eubacterium] nodatum group |
| OTU429 | 0.00(0.20, 0.00) | 0.00(0.01, 0.00) | 0.83 | shared | Firmicutes | Ruminococcaceae | Ruminococcaceae UCG-010 |
| OTU431 | 0.00(0.00, 0.00) | 0.05(0.12, 0.00) | 0.02 | C.unique | Firmicutes | Erysipelotrichaceae | Erysipelotrichaceae UCG-009 |
| OTU433 | 0.04(0.06, 0.01) | 0.02(0.11, 0.00) | 0.6 | shared | Firmicutes | Ruminococcaceae | Ruminococcaceae NK4A214 group |
| OTU434 | 0.02(0.14, 0.00) | 0.00(0.01, 0.00) | 0.46 | shared | Firmicutes | Ruminococcaceae | Ruminococcaceae UCG-010 |
| OTU437 | 0.00(0.02, 0.00) | 0.04(0.16, 0.00) | 0.03 | shared | Firmicutes | Lachnospiraceae | Coprococcus 1 |
| OTU438 | 0.00(0.03, 0.00) | 0.02(0.14, 0.00) | 0.13 | shared | Firmicutes | Ruminococcaceae | Ruminococcaceae UCG-014 |
| OTU439 | 0.04(0.62, 0.01) | 0.00(0.04, 0.00) | 0.08 | shared | Firmicutes | Ruminococcaceae | [Eubacterium] coprostanoligenes group |
| OTU444 | 0.01(0.17, 0.00) | 0.00(0.03, 0.00) | 0.32 | shared | Bacteroidetes | Bacteroidales Incertae Sedis | Phocaeicola |
| OTU445 | 0.00(0.21, 0.00) | 0.01(0.02, 0.00) | 0.92 | shared | Proteobacteria | Enterobacteriaceae | Escherichia-Shigella |
| OTU446 | 0.06(0.10, 0.00) | 0.00(0.01, 0.00) | 0.13 | shared | Firmicutes | Ruminococcaceae | Ruminococcaceae UCG-010 |
| OTU450 | 0.00(0.00, 0.00) | 0.03(0.13, 0.00) | 0.05 | C.unique | Firmicutes | Erysipelotrichaceae | Erysipelotrichaceae UCG-006 |
| OTU451 | 0.26(0.53, 0.11) | 0.07(1.07, 0.00) | 0.12 | shared | Firmicutes | Ruminococcaceae | Ruminococcaceae UCG-005 |
| OTU454 | 0.11(0.15, 0.05) | 0.21(0.53, 0.01) | 0.12 | shared | Firmicutes | Lachnospiraceae | Lachnoclostridium 5 |
| OTU456 | 0.00(0.00, 0.00) | 0.01(0.11, 0.00) | 0.02 | C.unique | Actinobacteria | Coriobacteriaceae | Parvibacter |
| OTU457 | 0.00(0.00, 0.00) | 0.00(0.14, 0.00) | 0.64 | shared | Firmicutes | Ruminococcaceae | Ruminococcaceae UCG-010 |
| OTU458 | 0.03(0.05, 0.00) | 0.14(0.38, 0.01) | 0.25 | shared | Firmicutes | Ruminococcaceae | Ruminococcaceae UCG-014 |
| OTU461 | 0.00(0.00, 0.00) | 0.05(0.16, 0.00) | 0.05 | C.unique | Firmicutes | Ruminococcaceae | Candidatus Soleaferrea |
| OTU465 | 0.00(0.00, 0.00) | 0.09(0.18, 0.00) | < 0.01 | C.unique | Actinobacteria | Nakamurella | Nakamurella |
| OTU466 | 0.00(0.17, 0.00) | 0.00(0.01, 0.00) | 0.44 | shared | Firmicutes | Ruminococcaceae | Ruminococcaceae UCG-010 |
| OTU468 | 0.00(0.33, 0.00) | 0.00(0.03, 0.00) | 0.73 | shared | Bacteroidetes | Porphyromonadaceae | Barnesiella |
| OTU470 | 0.01(0.11, 0.00) | 0.00(0.00, 0.00) | 0.05 | H.unique | Proteobacteria | Desulfovibrionaceae | Desulfovibrio |
| OTU472 | 0.00(0.21, 0.00) | 0.00(0.00, 0.00) | 0.14 | H.unique | Bacteroidetes | Prevotellaceae | Prevotellaceae UCG-004 |
| OTU474 | 0.00(0.00, 0.00) | 0.00(0.21, 0.00) | 0.14 | C.unique | Firmicutes | Ruminococcaceae | Ruminococcus 1 |
| OTU478 | 0.01(0.13, 0.00) | 0.02(0.09, 0.00) | 0.6 | shared | Firmicutes | Clostridiales vadinBB60 group | Christensenella |
| OTU482 | 0.05(0.14, 0.00) | 0.03(1.15, 0.01) | 0.75 | shared | Euryarchaeota | Methanobacteriaceae | Methanobrevibacter |
| OTU483 | 0.01(0.21, 0.00) | 0.04(0.05, 0.00) | 0.92 | shared | Firmicutes | Ruminococcaceae | Ruminococcaceae UCG-010 |
| OTU485 | 0.00(0.01, 0.00) | 0.00(0.19, 0.00) | 0.64 | shared | Firmicutes | Ruminococcaceae | Ruminococcaceae UCG-010 |
| OTU489 | 0.00(0.16, 0.00) | 0.01(0.04, 0.00) | 0.92 | shared | Firmicutes | Ruminococcaceae | Ruminococcus |
| OTU497 | 0.00(0.41, 0.00) | 0.05(0.60, 0.01) | 0.24 | shared | Verrucomicrobia | Verrucomicrobiaceae | Akkermansia |
| OTU503 | 0.00(0.12, 0.00) | 0.02(0.07, 0.00) | 0.75 | shared | Firmicutes | Ruminococcaceae | Ruminococcaceae UCG-010 |
| OTU504 | 0.00(0.01, 0.00) | 0.00(0.17, 0.00) | 0.19 | shared | Firmicutes | Ruminococcaceae | Ruminococcaceae UCG-014 |
| OTU506 | 0.01(0.03, 0.01) | 0.06(0.13, 0.03) | < 0.01 | shared | Firmicutes | Family XIII | Mogibacterium |
| OTU510 | 0.00(0.12, 0.00) | 0.00(0.08, 0.00) | 0.81 | shared | Tenericutes | Spiroplasmataceae | Spiroplasma |
| OTU511 | 0.00(0.00, 0.00) | 0.00(0.17, 0.00) | 0.14 | C.unique | Firmicutes | Erysipelotrichaceae | Erysipelotrichaceae UCG-009 |
| OTU524 | 0.00(0.15, 0.00) | 0.01(0.06, 0.00) | 0.28 | shared | Firmicutes | Ruminococcaceae | Ruminococcaceae UCG-010 |
| OTU528 | 0.00(0.00, 0.00) | 0.00(0.14, 0.00) | 0.14 | C.unique | Proteobacteria | Rhodospirillaceae | uncultured |
| OTU529 | 0.66(0.99, 0.26) | 0.26(0.56, 0.02) | 0.12 | shared | Firmicutes | Ruminococcaceae | Ruminococcaceae UCG-005 |
| OTU532 | 0.00(0.01, 0.00) | 0.00(0.10, 0.00) | 0.88 | shared | Actinobacteria | Kineococcus | Kineococcus |
| OTU535 | 0.00(0.00, 0.00) | 0.01(0.13, 0.00) | 0.02 | C.unique | Actinobacteria | Coriobacteriaceae | Senegalimassilia |
| OTU539 | 0.00(0.01, 0.00) | 0.01(0.14, 0.00) | 0.22 | shared | Firmicutes | Ruminococcaceae | Ruminococcus |
| OTU542 | 0.07(0.73, 0.01) | 0.00(0.01, 0.00) | 0.02 | shared | Firmicutes | Christensenellaceae | Christensenellaceae R-7 group |
| OTU549 | 0.03(0.04, 0.00) | 0.02(0.11, 0.00) | 0.68 | shared | Firmicutes | Lachnospiraceae | Dorea |
| OTU552 | 0.00(0.02, 0.00) | 0.01(0.14, 0.00) | 0.07 | shared | Firmicutes | Lachnospiraceae | Acetitomaculum |
| OTU557 | 0.00(0.01, 0.00) | 0.04(0.11, 0.00) | 0.13 | shared | Actinobacteria | Herbidospora | Herbidospora |
| OTU558 | 0.00(0.00, 0.00) | 0.01(0.10, 0.00) | 0.02 | C.unique | Actinobacteria | Occallatibacter | Occallatibacter |
| OTU562 | 0.00(0.11, 0.00) | 0.00(0.00, 0.00) | 0.14 | H.unique | Bacteroidetes | Prevotellaceae | Prevotella 1 |
| OTU567 | 0.00(0.16, 0.00) | 0.00(0.00, 0.00) | 0.14 | H.unique | Bacteroidetes | Bacteroidales S24-7 group | Muribaculum |
| OTU579 | 0.27(0.52, 0.09) | 0.09(0.16, 0.00) | 0.05 | shared | Firmicutes | Ruminococcaceae | Ruminococcaceae UCG-005 |
| OTU580 | 0.00(0.00, 0.00) | 0.00(0.17, 0.00) | 0.14 | C.unique | Bacteroidetes | Rikenellaceae | Rikenellaceae RC9 gut group |
| OTU582 | 0.04(0.20, 0.00) | 0.00(0.00, 0.00) | 0.02 | H.unique | Firmicutes | Ruminococcaceae | Ruminococcaceae UCG-010 |
| OTU586 | 0.01(0.03, 0.00) | 0.15(0.27, 0.02) | 0.03 | shared | Saccharibacteria | Unknown Family | Candidatus Saccharimonas |
| OTU599 | 0.00(0.00, 0.00) | 0.00(0.15, 0.00) | 0.14 | C.unique | Proteobacteria | Succinivibrionaceae | Succinivibrionaceae UCG-002 |
| OTU602 | 0.00(0.00, 0.00) | 0.02(0.12, 0.00) | < 0.01 | C.unique | Firmicutes | Erysipelotrichaceae | Holdemania |
| OTU607 | 0.00(0.01, 0.00) | 0.02(0.13, 0.01) | 0.02 | shared | Firmicutes | Christensenellaceae | Christensenellaceae R-7 group |
| OTU608 | 0.04(0.11, 0.00) | 0.01(0.06, 0.00) | 0.53 | shared | Firmicutes | Ruminococcaceae | Ruminococcus |
| OTU610 | 0.09(0.15, 0.03) | 0.01(0.03, 0.00) | < 0.01 | shared | Firmicutes | Ruminococcaceae | Ruminococcaceae UCG-005 |
| OTU613 | 0.00(0.00, 0.00) | 0.01(0.12, 0.00) | 0.05 | shared | Firmicutes | Ruminococcaceae | [Eubacterium] coprostanoligenes group |
| OTU616 | 0.10(0.18, 0.00) | 0.01(0.01, 0.00) | 0.07 | shared | Firmicutes | Ruminococcaceae | [Eubacterium] coprostanoligenes group |
| OTU620 | 0.00(0.11, 0.00) | 0.00(0.00, 0.00) | 0.14 | H.unique | Bacteroidetes | Bacteroidales Incertae Sedis | Phocaeicola |
| OTU638 | 0.02(0.12, 0.00) | 0.01(0.04, 0.00) | 0.45 | shared | Firmicutes | Ruminococcaceae | Ruminococcaceae UCG-013 |
| OTU640 | 0.00(0.00, 0.00) | 0.00(0.13, 0.00) | 0.14 | C.unique | Firmicutes | Lachnospiraceae | Lachnospiraceae NK3A20 group |
| OTU642 | 0.00(0.14, 0.00) | 0.03(0.04, 0.01) | 0.11 | shared | Firmicutes | Ruminococcaceae | Ruminococcus 2 |
| OTU675 | 0.00(0.00, 0.00) | 0.01(0.15, 0.00) | < 0.01 | C.unique | Firmicutes | Christensenellaceae | Christensenellaceae R-7 group |
| OTU676 | 0.00(0.00, 0.00) | 0.17(0.58, 0.12) | < 0.01 | C.unique | Saccharibacteria | Unknown Family | Candidatus Saccharimonas |
| OTU683 | 0.01(0.12, 0.01) | 0.00(0.00, 0.00) | < 0.01 | shared | Bacteroidetes | Porphyromonadaceae | Barnesiella |
| OTU686 | 0.00(0.00, 0.00) | 0.00(0.10, 0.00) | 0.05 | C.unique | Bacteroidetes | Prevotellaceae | Prevotellaceae UCG-004 |
| OTU690 | 0.03(0.09, 0.01) | 0.05(0.16, 0.03) | 0.17 | shared | Firmicutes | Family XIII | Family XIII AD3011 group |
| OTU718 | 0.00(0.00, 0.00) | 0.04(0.49, 0.01) | < 0.01 | C.unique | Firmicutes | Family XIII | [Eubacterium] nodatum group |
| OTU729 | 0.00(0.01, 0.00) | 0.01(0.11, 0.00) | 0.06 | shared | Firmicutes | Ruminococcaceae | Ruminococcaceae UCG-014 |
| OTU740 | 0.02(0.11, 0.01) | 0.00(0.00, 0.00) | < 0.01 | H.unique | Spirochaetae | Spirochaetaceae | Treponema 2 |
| OTU774 | 0.00(0.01, 0.00) | 0.02(0.24, 0.00) | 0.03 | shared | Actinobacteria | Coriobacteriaceae | Senegalimassilia |
| OTU789 | 0.00(0.01, 0.00) | 0.01(0.11, 0.00) | 0.06 | shared | Firmicutes | Clostridiaceae 1 | Clostridium sensu stricto 1 |
| OTU816 | 0.02(0.11, 0.01) | 0.00(0.01, 0.00) | < 0.01 | shared | Firmicutes | Lachnospiraceae | Lachnoclostridium |
| OTU822 | 0.00(0.00, 0.00) | 0.00(0.11, 0.00) | 0.03 | C.unique | Firmicutes | Ruminococcaceae | Ruminococcaceae NK4A214 group |
| OTU841 | 0.04(1.41, 0.00) | 0.03(0.09, 0.01) | 0.6 | shared | Bacteroidetes | Bacteroidaceae | Bacteroides |
| OTU856 | 0.11(2.23, 0.07) | 0.00(0.01, 0.00) | < 0.01 | shared | Firmicutes | Ruminococcaceae | [Eubacterium] coprostanoligenes group |
| OTU890 | 0.94(2.15, 0.63) | 0.02(0.03, 0.00) | < 0.01 | shared | Firmicutes | Christensenellaceae | Christensenellaceae R-7 group |
| OTU902 | 0.05(0.10, 0.01) | 0.00(0.01, 0.00) | < 0.01 | shared | Firmicutes | Ruminococcaceae | Ruminococcaceae UCG-005 |
| OTU923 | 0.00(0.01, 0.00) | 0.06(0.27, 0.00) | 0.02 | shared | Firmicutes | Lachnospiraceae | Syntrophococcus |
| OTU930 | 0.02(0.08, 0.00) | 0.00(0.11, 0.00) | 0.73 | shared | Firmicutes | Ruminococcaceae | Ruminococcaceae UCG-014 |
| OTU932 | 0.00(0.01, 0.00) | 0.03(0.21, 0.01) | < 0.01 | shared | Actinobacteria | Coriobacteriaceae | Senegalimassilia |
| OTU940 | 0.00(0.00, 0.00) | 0.00(0.20, 0.00) | 0.7 | C.unique | Firmicutes | Ruminococcaceae | Ruminiclostridium 6 |
| OTU956 | 0.00(0.00, 0.00) | 0.00(0.14, 0.00) | 0.14 | C.unique | Actinobacteria | Coriobacteriaceae | Senegalimassilia |
| OTU960 | 0.00(0.00, 0.00) | 0.00(0.11, 0.00) | 0.05 | C.unique | Bacteroidetes | Rikenellaceae | Rikenellaceae RC9 gut group |
| OTU975 | 0.29(0.52, 0.14) | 0.03(0.07, 0.01) | < 0.01 | shared | Firmicutes | Lachnospiraceae | Lachnospiraceae NK4A136 group |
| OTU998 | 0.00(0.00, 0.00) | 0.06(0.28, 0.01) | < 0.01 | C.unique | Firmicutes | Ruminococcaceae | Ruminococcaceae NK4A214 group |
| OTU1010 | 0.02(0.05, 0.01) | 0.28(0.40, 0.01) | 0.17 | shared | Firmicutes | Christensenellaceae | Christensenellaceae R-7 group |
| OTU1025 | 0.02(0.10, 0.00) | 0.18(1.36, 0.11) | < 0.01 | shared | Firmicutes | Ruminococcaceae | Ruminococcaceae UCG-014 |
| OTU1028 | 0.00(0.00, 0.00) | 0.03(0.17, 0.00) | 0.05 | C.unique | Actinobacteria | Streptosporangium | Streptosporangium |
| OTU1039 | 0.00(0.00, 0.00) | 0.17(0.58, 0.00) | 0.05 | C.unique | Firmicutes | Christensenellaceae | Christensenellaceae R-7 group |
| OTU1063 | 0.01(0.19, 0.00) | 0.01(0.03, 0.00) | 0.68 | shared | Bacteroidetes | Rikenellaceae | Rikenellaceae RC9 gut group |
| OTU1071 | 0.06(0.61, 0.00) | 0.01(0.04, 0.00) | 0.2 | shared | Firmicutes | Christensenellaceae | Christensenellaceae R-7 group |
| OTU1099 | 0.00(0.00, 0.00) | 0.04(0.28, 0.00) | 0.02 | C.unique | Firmicutes | Lachnospiraceae | [Ruminococcus] gauvreauii group |
| OTU1109 | 0.00(0.23, 0.00) | 0.00(0.01, 0.00) | 0.28 | shared | Firmicutes | Family XIII | [Eubacterium] nodatum group |
| OTU1118 | 0.02(0.12, 0.00) | 0.00(0.01, 0.00) | 0.05 | shared | Firmicutes | Christensenellaceae | Christensenellaceae R-7 group |
| OTU1133 | 0.00(0.02, 0.00) | 0.54(1.80, 0.19) | < 0.01 | shared | Saccharibacteria | Unknown Family | Candidatus Saccharimonas |
| OTU1163 | 0.00(0.00, 0.00) | 0.00(0.13, 0.00) | 0.14 | C.unique | Cyanobacteria | Calochaete | Calochaete |
| OTU1171 | 0.09(0.14, 0.02) | 0.06(0.12, 0.04) | 0.92 | shared | Firmicutes | Christensenellaceae | Christensenellaceae R-7 group |
| OTU1174 | 0.00(0.00, 0.00) | 0.01(0.12, 0.00) | 0.02 | C.unique | Firmicutes | Lachnospiraceae | Lachnospiraceae NK3A20 group |
| OTU1182 | 0.00(0.36, 0.00) | 0.00(0.04, 0.00) | 0.52 | shared | Firmicutes | Clostridiales vadinBB60 group | Christensenella |
| OTU1193 | 0.00(0.00, 0.00) | 0.67(0.97, 0.08) | < 0.01 | C.unique | Saccharibacteria | Unknown Family | Candidatus Saccharimonas |
| OTU1195 | 0.07(0.22, 0.00) | 0.06(0.23, 0.00) | 0.92 | shared | Firmicutes | Ruminococcaceae | Ruminococcaceae UCG-014 |
| OTU1200 | 0.01(0.06, 0.00) | 0.11(0.33, 0.05) | 0.02 | shared | Firmicutes | Family XIII | Mogibacterium |
| OTU1203 | 0.00(0.00, 0.00) | 0.00(0.16, 0.00) | 0.14 | C.unique | Firmicutes | Eubacteriaceae | Alkalibaculum |
| OTU1220 | 0.00(0.00, 0.00) | 0.02(0.26, 0.00) | 0.05 | C.unique | Firmicutes | Lachnospiraceae | Syntrophococcus |
| OTU1222 | 0.00(0.00, 0.00) | 0.08(0.31, 0.00) | 0.03 | C.unique | Cyanobacteria | Iphinoe | Iphinoe |
| OTU1234 | 0.00(0.00, 0.00) | 0.00(0.22, 0.00) | 0.05 | C.unique | Firmicutes | Christensenellaceae | Christensenellaceae R-7 group |
| OTU1241 | 0.00(0.00, 0.00) | 0.00(0.35, 0.00) | 0.02 | C.unique | Saccharibacteria | Unknown Family | Candidatus Saccharimonas |
| OTU1243 | 0.05(0.11, 0.01) | 0.08(0.55, 0.01) | 0.35 | shared | Firmicutes | Family XIII | [Eubacterium] nodatum group |
| OTU1272 | 0.00(0.00, 0.00) | 0.03(0.17, 0.00) | 0.02 | C.unique | Saccharibacteria | Unknown Family | Candidatus Saccharimonas |
| OTU1284 | 0.11(0.12, 0.00) | 0.04(0.06, 0.00) | 0.29 | shared | Firmicutes | Christensenellaceae | Christensenellaceae R-7 group |
| OTU1285 | 0.00(0.01, 0.00) | 0.00(0.14, 0.00) | 0.92 | shared | Firmicutes | Ruminococcaceae | Ruminococcaceae UCG-010 |
| OTU1307 | 0.04(0.25, 0.01) | 0.04(0.11, 0.00) | 0.92 | shared | Verrucomicrobia | Verrucomicrobiaceae | Akkermansia |
| OTU1319 | 0.00(0.00, 0.00) | 0.00(0.11, 0.00) | 0.05 | C.unique | Firmicutes | Ruminococcaceae | [Eubacterium] coprostanoligenes group |
| OTU1326 | 0.05(0.12, 0.03) | 0.00(0.17, 0.00) | 0.12 | shared | Firmicutes | Ruminococcaceae | [Eubacterium] coprostanoligenes group |
| OTU1327 | 0.00(0.00, 0.00) | 0.04(0.15, 0.00) | 0.03 | C.unique | Firmicutes | Ruminococcaceae | Ruminococcaceae UCG-014 |
| OTU1332 | 0.13(0.16, 0.02) | 0.24(0.49, 0.05) | 0.25 | shared | Firmicutes | Christensenellaceae | Christensenellaceae R-7 group |
| OTU1347 | 0.00(0.13, 0.00) | 0.00(0.01, 0.00) | 0.52 | shared | Firmicutes | Ruminococcaceae | Ruminococcaceae UCG-014 |
| OTU1348 | 0.00(0.00, 0.00) | 0.08(0.18, 0.06) | < 0.01 | C.unique | Firmicutes | Christensenellaceae | Christensenellaceae R-7 group |
| OTU1361 | 0.01(0.03, 0.00) | 0.10(0.17, 0.01) | 0.05 | shared | Firmicutes | Christensenellaceae | Christensenellaceae R-7 group |
| OTU1379 | 0.00(0.23, 0.00) | 0.28(0.86, 0.15) | 0.03 | shared | Firmicutes | Christensenellaceae | Christensenellaceae R-7 group |
| OTU1388 | 0.00(0.00, 0.00) | 0.00(0.27, 0.00) | 0.05 | C.unique | Firmicutes | Caldicoprobacteraceae | Caldicoprobacter |
| OTU1413 | 0.33(0.78, 0.01) | 0.02(0.09, 0.01) | 0.12 | shared | Firmicutes | Christensenellaceae | Christensenellaceae R-7 group |
| OTU1429 | 0.00(0.00, 0.00) | 0.00(0.19, 0.00) | 0.14 | C.unique | Actinobacteria | Coriobacteriaceae | Senegalimassilia |
| OTU1445 | 0.00(0.01, 0.00) | 0.00(0.14, 0.00) | 0.19 | shared | Firmicutes | Ruminococcaceae | Ruminococcaceae UCG-014 |
| OTU1455 | 0.12(0.34, 0.01) | 0.01(0.02, 0.00) | 0.08 | shared | Firmicutes | Christensenellaceae | Christensenellaceae R-7 group |
| OTU1457 | 0.00(0.00, 0.00) | 0.01(0.21, 0.00) | 0.05 | C.unique | Saccharibacteria | Unknown Family | Candidatus Saccharimonas |
| OTU1460 | 0.00(0.22, 0.00) | 0.01(0.03, 0.00) | 0.75 | shared | Firmicutes | Christensenellaceae | Christensenellaceae R-7 group |
| OTU1472 | 0.04(0.06, 0.01) | 0.13(0.19, 0.01) | 0.08 | shared | Firmicutes | Christensenellaceae | Christensenellaceae R-7 group |
| OTU1476 | 0.03(0.17, 0.00) | 0.00(0.02, 0.00) | 0.22 | shared | Bacteroidetes | Bacteroidales BS11 gut group | Pseudopedobacter |
| OTU1492 | 0.03(0.06, 0.00) | 0.09(0.21, 0.01) | 0.08 | shared | Firmicutes | Christensenellaceae | Christensenellaceae R-7 group |
| OTU1496 | 0.03(0.09, 0.00) | 0.04(0.12, 0.00) | 0.92 | shared | Firmicutes | Clostridiales vadinBB60 group | Christensenella |
| OTU1501 | 0.00(0.37, 0.00) | 0.01(0.05, 0.00) | 0.22 | shared | Firmicutes | Christensenellaceae | Christensenellaceae R-7 group |
| OTU1512 | 0.00(0.00, 0.00) | 0.16(0.72, 0.08) | < 0.01 | C.unique | Saccharibacteria | Unknown Family | Candidatus Saccharimonas |
| OTU1526 | 0.00(0.01, 0.00) | 0.07(0.10, 0.01) | 0.02 | shared | Firmicutes | Family XIII | Family XIII AD3011 group |
| OTU1529 | 0.00(0.01, 0.00) | 0.00(0.12, 0.00) | 0.28 | shared | Firmicutes | Ruminococcaceae | Ruminococcus 2 |
| OTU1537 | 0.00(0.00, 0.00) | 0.02(0.10, 0.00) | 0.53 | C.unique | Actinobacteria | Coriobacteriaceae | Olsenella |
| OTU1559 | 0.00(0.00, 0.00) | 0.16(0.30, 0.02) | < 0.01 | C.unique | Firmicutes | Christensenellaceae | Christensenellaceae R-7 group |
| OTU1566 | 0.01(0.06, 0.00) | 0.06(0.18, 0.02) | 0.05 | shared | Firmicutes | Christensenellaceae | Christensenellaceae R-7 group |
| OTU1572 | 0.00(0.00, 0.00) | 0.05(0.13, 0.02) | < 0.01 | C.unique | Firmicutes | Christensenellaceae | Christensenellaceae R-7 group |
| OTU1573 | 0.00(0.20, 0.00) | 0.00(0.00, 0.00) | 0.37 | H.unique | Firmicutes | Christensenellaceae | Christensenellaceae R-7 group |
| OTU1589 | 0.03(5.95, 0.01) | 0.00(0.01, 0.00) | < 0.01 | shared | Firmicutes | Ruminococcaceae | Ruminococcaceae UCG-005 |
| OTU1596 | 0.00(0.06, 0.00) | 0.02(0.11, 0.00) | 0.24 | shared | Firmicutes | Christensenellaceae | Christensenellaceae R-7 group |
| OTU1614 | 0.00(0.01, 0.00) | 0.07(0.29, 0.00) | 0.06 | shared | Actinobacteria | Modestobacter | Modestobacter |
| OTU1622 | 0.00(0.00, 0.00) | 0.01(0.25, 0.01) | < 0.01 | C.unique | Actinobacteria | Coriobacteriaceae | Olsenella |
| OTU1624 | 0.98(1.66, 0.29) | 0.48(1.27, 0.05) | 0.25 | shared | Firmicutes | Ruminococcaceae | Ruminococcaceae UCG-005 |
| OTU1647 | 0.00(0.03, 0.00) | 0.05(0.12, 0.01) | 0.03 | shared | Firmicutes | Lachnospiraceae | Marvinbryantia |
| OTU1669 | 0.01(0.07, 0.00) | 0.06(0.12, 0.01) | 0.12 | shared | Firmicutes | Lachnospiraceae | [Ruminococcus] gauvreauii group |
| OTU1679 | 0.11(0.23, 0.03) | 0.01(0.05, 0.00) | 0.03 | shared | Firmicutes | Ruminococcaceae | Ruminococcaceae UCG-005 |
| OTU1683 | 0.00(0.01, 0.00) | 0.09(0.28, 0.01) | < 0.01 | shared | Acidobacteria | Occallatibacter | Occallatibacter |
| OTU1687 | 0.83(1.07, 0.05) | 0.65(1.26, 0.09) | 0.75 | shared | Firmicutes | Ruminococcaceae | Ruminococcaceae UCG-005 |
| OTU1740 | 0.00(0.00, 0.00) | 0.01(0.12, 0.01) | < 0.01 | C.unique | Firmicutes | Christensenellaceae | Christensenellaceae R-7 group |
| OTU1741 | 0.05(0.11, 0.00) | 0.03(0.28, 0.00) | 0.75 | shared | Firmicutes | Ruminococcaceae | Ruminococcaceae UCG-005 |
| OTU1744 | 0.08(0.28, 0.00) | 0.02(0.15, 0.01) | 0.75 | shared | Firmicutes | Ruminococcaceae | Ruminococcaceae UCG-014 |
| OTU1761 | 0.00(0.44, 0.00) | 0.06(0.20, 0.00) | 0.28 | shared | Firmicutes | Ruminococcaceae | Ruminococcus 2 |

^a^ abundance value are expressed as medians(maximum, minimum), n=5 lactating goats/group;

Mann-Whitney U test *P* value was used; LC, low concentration diet; HC, high concentration diet; OTU, operational taxonomic unit.
