# Supplementary material for: Microbial community shifts elicit inflammation in the caecal mucosa via the GPR41/43 signalling pathway during subacute ruminal acidosis
Source: BMC Vet Res. 2019 Aug 19;15:298. doi: 10.1186/s12917-019-2031-5 (PMC6700796; doi:10.1186/s12917-019-2031-5)
Supplement: Supplementary file 6 — Table S6. Relative abundance of bacterial phyla in cecal content of goats from LC group or HC group. (DOCX 14 kb) [file 12917_2019_2031_MOESM6_ESM.docx]

Table S6. Relative abundance of bacterial phyla in cecal content of goats from LC group or HC group. Only phyla which relative abundances were greater than 0.5% in at least one sample,were listed.

| Phyla(%) | LC^a^ | HC^a^ | *p* value |
| --- | --- | --- | --- |
| Firmicutes | 70.13(88.73, 61.32) | 56.57(59.07, 38.55) | <0.01 |
| Bacteroidetes | 15.97(25.82, 2.48) | 1.78(5.39, 0.03) | 0.02 |
| Euryarchaeota | 1.94(10.16, 1.56) | 9.46(25.67, 3.70) | 0.04 |
| Actinobacteria | 0.82(0.99, 0.16) | 5.78(24.85, 4.00) | <0.01 |
| Verrucomicrobia | 2.44(15.2, 1.56) | 0.69(3.39, 0.13) | 0.04 |
| Proteobacteria | 0.60(0.81, 0.23) | 4.80(8.43, 1.47) | <0.01 |
| Cyanobacteria | 0.10(0.27, 0.01) | 3.86(5.65, 1.58) | <0.01 |
| Spirochaetae | 0.28(2.31, 0.13) | 0.12(1.90, 0.03) | 0.25 |
| Tenericutes | 0.45(0.76, 0.13) | 0.44(0.76, 0.12) | 0.83 |
| Others | 0.06(1.22, 0.00) | 10.20(2.55, 15.32) | <0.01 |

^a^ abundance value are expressed as medians(maximum, minimum), n=5 lactating goats/group;

Mann-Whitney U test *P* value was used; LC, low concentration diet; HC, high concentration diet.
