# Supplementary material for: Microbial community shifts elicit inflammation in the caecal mucosa via the GPR41/43 signalling pathway during subacute ruminal acidosis
Source: BMC Vet Res. 2019 Aug 19;15:298. doi: 10.1186/s12917-019-2031-5 (PMC6700796; doi:10.1186/s12917-019-2031-5)
Supplement: Supplementary file 7 — Table S7. Relative abundance of bacterial genera in cecal content of lactating goats from LC or HC group. (DOCX 22 kb) [file 12917_2019_2031_MOESM7_ESM.docx]

Table S7 Relative abundance of bacterial genera in cecal content of lactating goats from LC or HC group. Only genus which relative abundance were more than 0.5% in at least one sample, were listed.

| Taxa(%) | LC^a^ | HC^a^ | *p* value | FC |
| --- | --- | --- | --- | --- |
| Acetitomaculum | 0.24(0.42, 0.03) | 1.24(2.83, 0.19) | 0.04 | 5 |
| Akkermansia | 2.44(15.2, 1.56) | 0.69(3.39, 0.13) | 0.04 | 4 |
| Alistipes | 0.84(1.52, 0.15) | 0.30(0.86, 0.00) | 0.25 | 3 |
| Anaerotruncus | 0.32(0.60, 0.23) | 0.16(1.56, 0.07) | 0.34 | 2 |
| Atopobium | 0.01(0.06, 0.00) | 0.29(1.61, 0.14) | <0.01 | 29 |
| Bacteroidales BS11 gut group_norank | 0.39(2.92, 0.00) | 0.09(0.17, 0.00) | 0.09 | 4 |
| Bacteroidales S24-7 group_norank | 2.43(4.98, 0.02) | 0.14(0.68, 0.01) | 0.04 | 17 |
| Bacteroidales UCG-001_norank | 0.11(3.54, 0.00) | 0.00(0.00, 0.00) | 0.02 | -- |
| Bacteroides | 1.28(9.65, 0.56) | 0.32(0.67, 0.01) | 0.02 | 4 |
| Barnesiella | 0.04(1.00, 0.01) | 0.01(0.03, 0.00) | 0.11 | 4 |
| Bifidobacteriaceae_uncultured | 0.00(0.15, 0.00) | 0.01(2.64, 0.00) | 0.59 | -- |
| Bifidobacterium | 0.00(0.00, 0.00) | 0.04(5.45, 0.00) | 0.04 | -- |
| Blautia | 0.08(0.12, 0.04) | 0.10(1.11, 0.03) | 0.46 | 1 |
| Candidatus Saccharimonas | 0.05(1.18, 0.00) | 14.66(15.32, 2.55) | <0.01 | 293 |
| Candidatus Soleaferrea | 0.20(1.63, 0.16) | 0.11(0.30, 0.00) | 0.05 | 2 |
| Clostridiales vadinBB60 group_norank | 0.65(2.20, 0.11) | 0.50(0.77, 0.01) | 0.34 | 1 |
| Clostridium sensu stricto 1 | 0.00(0.06, 0.00) | 0.34(2.74, 0.03) | 0.01 | -- |
| Coriobacteriaceae UCG-002 | 0.00(0.01, 0.00) | 0.39(0.84, 0.24) | <0.01 | -- |
| Coriobacteriaceae_uncultured | 0.06(0.12, 0.03) | 0.36(1.52, 0.28) | <0.01 | 6 |
| dgA-11 gut group | 0.02(1.64, 0.00) | 0.14(0.58, 0.00) | 0.25 | 7 |
| Enterorhabdus | 0.00(0.02, 0.00) | 0.20(0.66, 0.01) | 0.02 | -- |
| Erysipelotrichaceae_uncultured | 0.08(0.16, 0.01) | 0.22(0.50, 0.10) | 0.03 | 3 |
| [Eubacterium] brachy group | 0.03(0.07, 0.02) | 0.39(0.66, 0.17) | <0.01 | 13 |
| [Eubacterium] coprostanoligenes group | 5.97(7.64, 2.44) | 1.33(1.95, 0.89) | <0.01 | 4 |
| [Eubacterium] nodatum group | 0.10(0.29, 0.03) | 2.60(5.09, 0.42) | <0.01 | 26 |
| Family XIII AD3011 group | 0.67(1.05, 0.27) | 3.97(9.62, 3.73) | <0.01 | 6 |
| Flavobacteriaceae_uncultured | 0.00(0.98, 0.00) | 0.08(0.19, 0.00) | 0.75 | -- |
| Gastranaerophilales_norank | 0.10(0.27, 0.01) | 3.86(5.65, 1.58) | <0.01 | 39 |
| Halomonas | 4.33(7.35, 1.16) | 0.20(0.53, 0.11) | <0.01 | 22 |
| Intestinibacter | 0.02(0.49, 0.01) | 1.19(11.62, 0.69) | <0.01 | 60 |
| Lachnoclostridium 5 | 0.11(0.15, 0.05) | 0.21(0.53, 0.01) | 0.11 | 2 |
| Lachnospiraceae AC2044 group | 0.39(1.18, 0.25) | 0.01(0.06, 0.00) | <0.01 | 39 |
| Lachnospiraceae FCS020 group | 0.09(0.91, 0.06) | 0.00(0.01, 0.00) | <0.01 | -- |
| Lachnospiraceae FE2018 group | 0.02(0.18, 0.00) | 0.45(0.71, 0.21) | <0.01 | 23 |
| Lachnospiraceae NK3A20 group | 0.19(0.42, 0.00) | 0.83(5.00, 0.62) | <0.01 | 4 |
| Lachnospiraceae NK4A136 group | 0.47(1.24, 0.28) | 0.04(0.07, 0.01) | <0.01 | 12 |
| Lachnospiraceae UCG-002 | 0.06(0.15, 0.00) | 0.40(0.48, 0.27) | <0.01 | 7 |
| Lachnospiraceae_uncultured | 1.40(2.40, 0.55) | 1.72(6.53, 0.51) | 0.75 | 1 |
| Marvinbryantia | 0.04(0.05, 0.00) | 0.26(0.60, 0.07) | <0.01 | 7 |
| Methanobrevibacter | 1.72(10.09, 0.43) | 18.08(25.27, 2.89) | <0.01 | 5 |
| Methanocorpusculum | 0.04(1.13, 0.00) | 0.00(0.06, 0.00) | 0.09 | -- |
| Methanosphaera | 0.01(0.02, 0.00) | 0.41(0.74, 0.24) | <0.01 | 41 |
| Mogibacterium | 0.21(0.39, 0.14) | 1.89(2.52, 1.36) | <0.01 | 9 |
| Mollicutes RF9_norank | 0.17(0.49, 0.00) | 0.35(0.65, 0.12) | 0.25 | 2 |
| NB1-n_norank | 0.08(0.38, 0.03) | 0.04(0.16, 0.00) | 0.34 | 2 |
| Olsenella | 0.08(0.35, 0.02) | 0.57(3.08, 0.14) | 0.03 | 7 |
| Oscillibacter | 0.18(0.64, 0.11) | 0.01(0.05, 0.00) | <0.01 | 18 |
| Parabacteroides | 0.14(2.38, 0.00) | 0.01(0.07, 0.00) | <0.01 | 14 |
| Peptococcaceae_uncultured | 0.30(0.49, 0.11) | 0.04(0.08, 0.00) | 0.11 | 8 |
| Phascolarctobacterium | 0.07(0.53, 0.03) | 0.00(0.06, 0.00) | 0.02 | -- |
| Phocaeicola | 0.14(0.63, 0.02) | 0.04(0.10, 0.00) | 0.06 | 4 |
| Prevotella 1 | 0.17(6.08, 0.02) | 0.02(0.03, 0.00) | <0.01 | 9 |
| Prevotellaceae UCG-001 | 0.14(0.96, 0.04) | 0.00(0.03, 0.00) | 0.07 | -- |
| Prevotellaceae UCG-003 | 1.42(1.87, 0.10) | 0.00(0.01, 0.00) | 0.01 | -- |
| Prevotellaceae UCG-004 | 0.53(1.77, 0.03) | 0.17(0.62, 0.01) | <0.01 | 3 |
| Rhodospirillaceae_uncultured | 0.07(0.62, 0.03) | 0.06(0.37, 0.02) | 0.25 | 1 |
| Rikenellaceae RC9 gut group | 1.64(3.18, 0.15) | 0.41(0.53, 0.00) | 0.6 | 4 |
| Roseburia | 0.08(0.27, 0.03) | 1.26(1.85, 0.30) | <0.01 | 16 |
| Ruminiclostridium 6 | 0.13(0.55, 0.11) | 0.08(0.29, 0.00) | <0.01 | 2 |
| Ruminococcaceae NK4A214 group | 3.66(4.61, 1.49) | 0.65(1.10, 0.08) | 0.17 | 6 |
| Ruminococcaceae UCG-002 | 8.7(13.78, 3.58) | 0.32(1.49, 0.06) | <0.01 | 27 |
| Ruminococcaceae UCG-005 | 8.59(10.41, 5.8) | 3.75(8.84, 0.29) | <0.01 | 2 |
| Ruminococcaceae UCG-007 | 0.13(0.72, 0.06) | 0.01(0.07, 0.00) | 0.05 | 13 |
| Ruminococcaceae UCG-010 | 3.10(8.07, 1.88) | 0.94(2.83, 0.09) | 0.02 | 3 |
| Ruminococcaceae UCG-013 | 2.21(4.62, 1.12) | 0.09(0.18, 0.02) | 0.02 | 25 |
| Ruminococcaceae UCG-014 | 3.28(6.18, 1.08) | 3.93(9.15, 1.13) | 0.08 | 1 |
| Ruminococcaceae_Unclassified | 0.24(0.65, 0.03) | 0.08(0.10, 0.01) | 0.6 | 3 |
| Ruminococcaceae_uncultured | 1.31(4.79, 1.09) | 0.64(1.20, 0.06) | 0.07 | 2 |
| [Ruminococcus] gauvreauii group | 0.08(0.98, 0.01) | 0.46(1.12, 0.23) | 0.01 | 6 |
| Ruminococcus 1 | 0.36(0.58, 0.17) | 0.01(0.33, 0.01) | 0.07 | 36 |
| Ruminococcus 2 | 0.13(1.50, 0.09) | 1.34(2.07, 0.08) | <0.01 | 10 |
| Rummeliibacillus | 0.00(0.00, 0.00) | 0.29(0.48, 0.00) | 0.46 | -- |
| Saccharofermentans | 0.13(0.74, 0.00) | 0.23(1.45, 0.06) | 0.02 | 2 |
| Senegalimassilia | 0.32(0.50, 0.01) | 2.72(11.13, 1.20) | 0.91 | 9 |
| Syntrophococcus | 0.03(0.12, 0.01) | 0.46(0.77, 0.13) | <0.01 | 15 |
| Treponema 2 | 0.27(2.31, 0.13) | 0.11(1.90, 0.03) | 0.02 | 2 |
| Turicibacter | 0.02(0.16, 0.00) | 0.65(3.80, 0.04) | 0.25 | 33 |
| Tyzzerella 4 | 1.06(2.76, 0.17) | 0.10(0.20, 0.05) | 0.01 | 11 |
| Others | 2.14(2.72, 1.59) | 4.15(4.61, 3.54) | 0.03 | 2 |

^a^ abundance value are expressed as medians(maximum, minimum), n=5 lactating goats/group;

Mann-Whitney U test *P* value was used; LC, low concentration diet; HC, high concentration diet. FC, fold changes.
