# Supplementary figures and images for: Microbial community shifts elicit inflammation in the caecal mucosa via the GPR41/43 signalling pathway during subacute ruminal acidosis
Source: BMC Vet Res. 2019 Aug 19;15:298. doi: 10.1186/s12917-019-2031-5 (PMC6700796; doi:10.1186/s12917-019-2031-5)

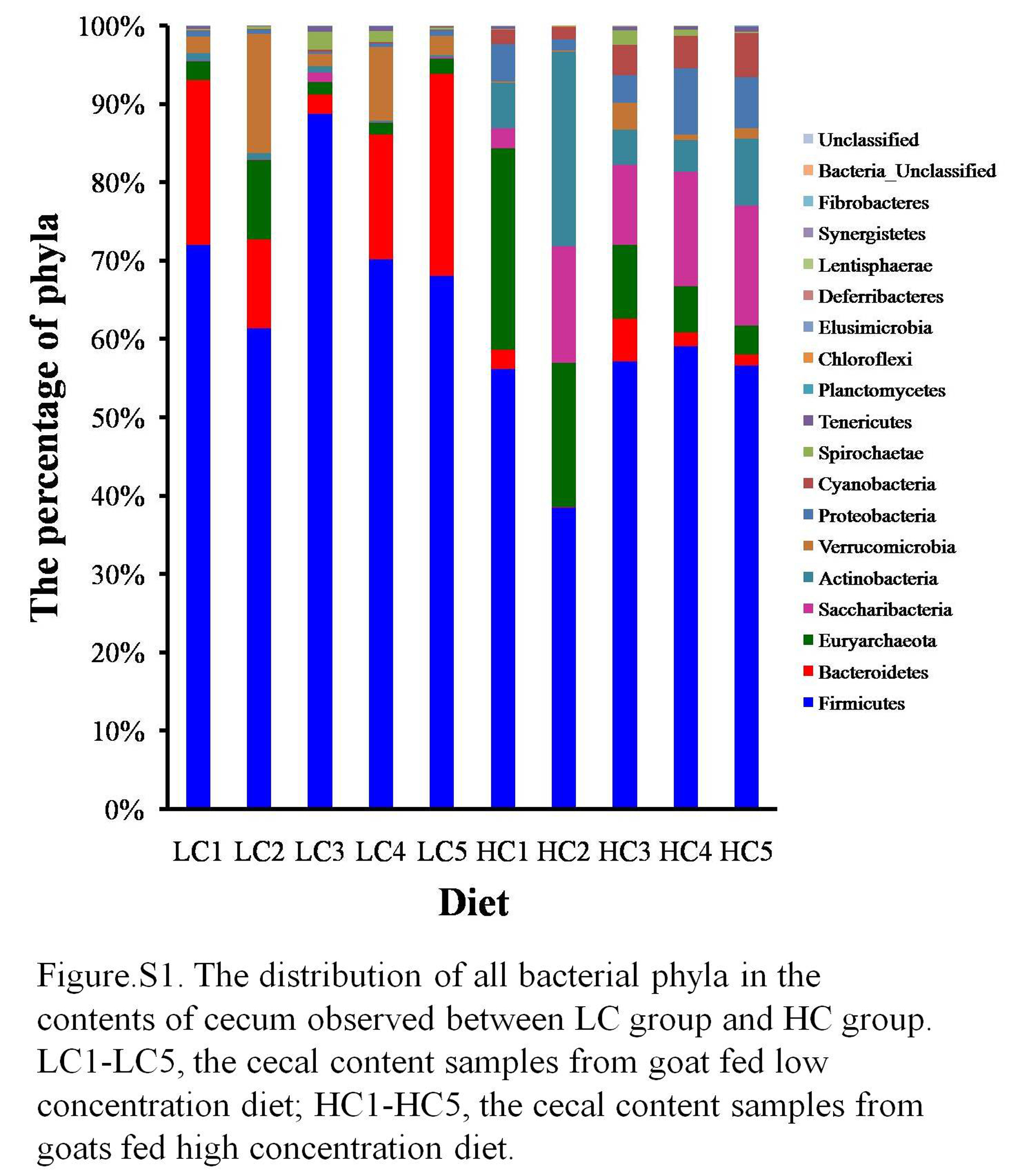

Supplement: Supplementary file 8 — Figure S1. The distribution of all bacterial phyla in the contents of cecum observed between LC group and HC group. LC1-LC5, the cecal content samples from goat fed low concentration diet; HC1-HC5, the cecal content samples from goats fed high concentration diet. (JPG 1308 kb) [file 12917_2019_2031_MOESM8_ESM.jpg]

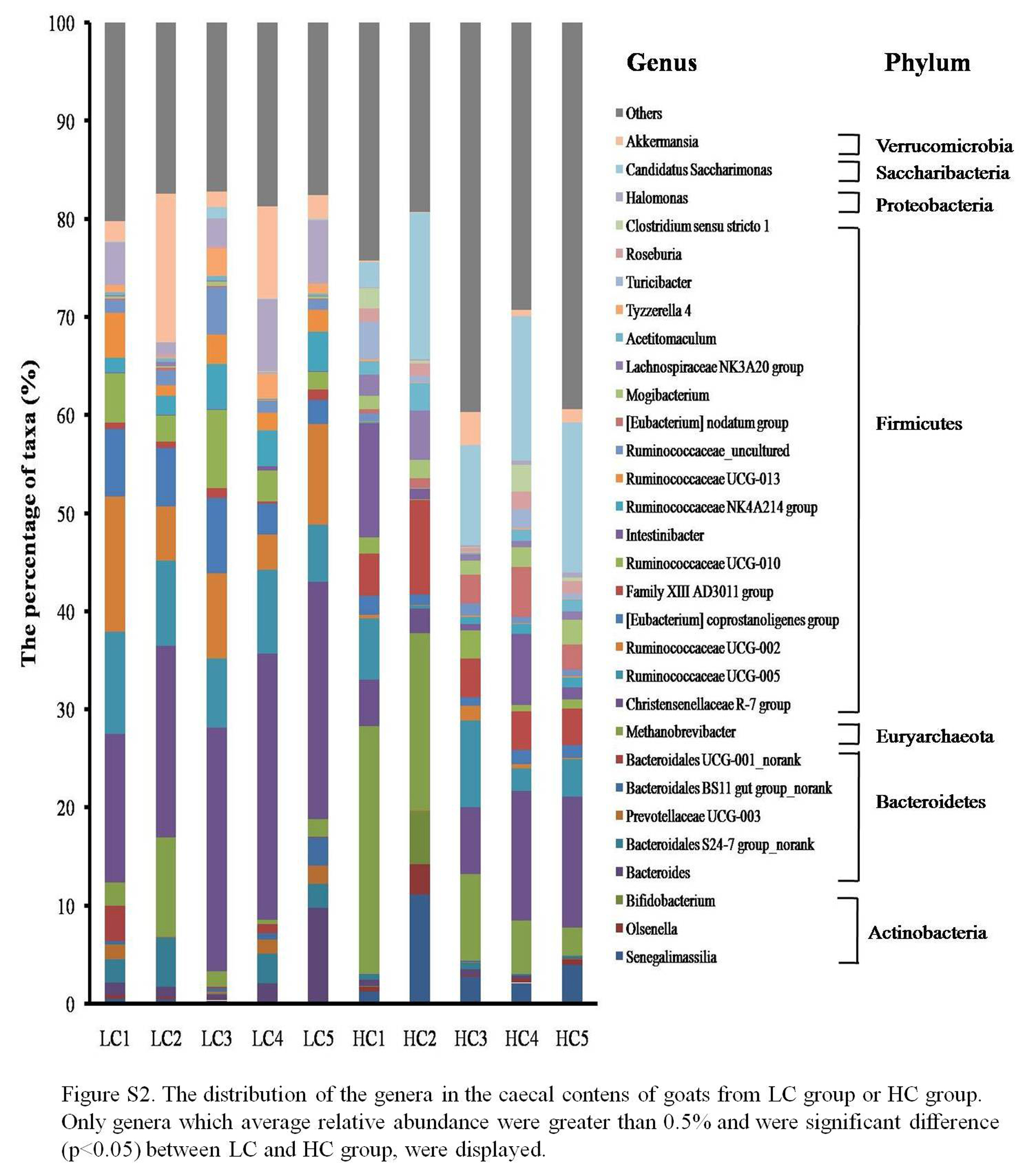

Supplement: Supplementary file 9 — Figure S2. The distribution of the genera in the caecal contens of goats from LC group or HC group. Only genera which average relative abundance were greater than 0.5% and were significant difference (p < 0.05) between LC and HC group, were displayed. (JPG 1237 kb) [file 12917_2019_2031_MOESM9_ESM.jpg]
