# Supplementary material for: Microbial community shifts elicit inflammation in the caecal mucosa via the GPR41/43 signalling pathway during subacute ruminal acidosis
Source: BMC Vet Res. 2019 Aug 19;15:298. doi: 10.1186/s12917-019-2031-5 (PMC6700796; doi:10.1186/s12917-019-2031-5)
Supplement: Supplementary file 10 — Western blotting image pictures. (DOCX 886 kb) [file 12917_2019_2031_MOESM10_ESM.docx]

**Western blotting image pictures**

TLR4 band picture


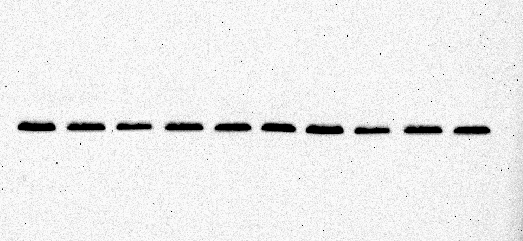


GPR41 band picture


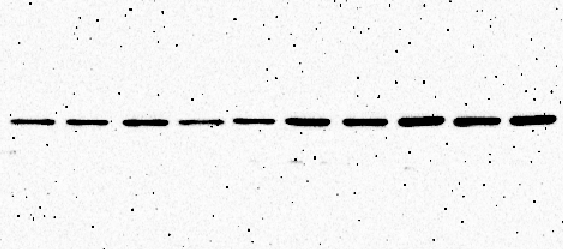


GPR43 band picture

**
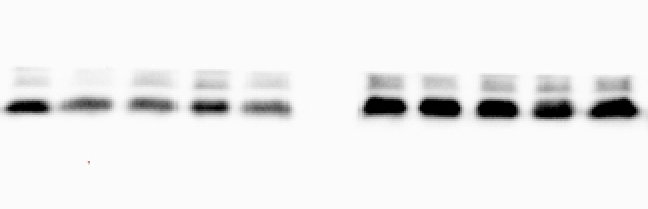
**

NF-κB p65 band picture

**
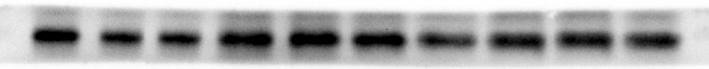
**

β-Tubulin band picture

**
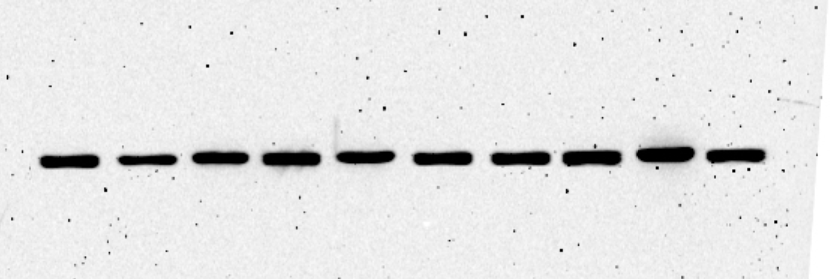
**

p38 band picture

**
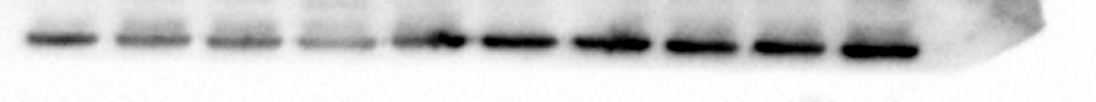
**

ERK1/2 band picture


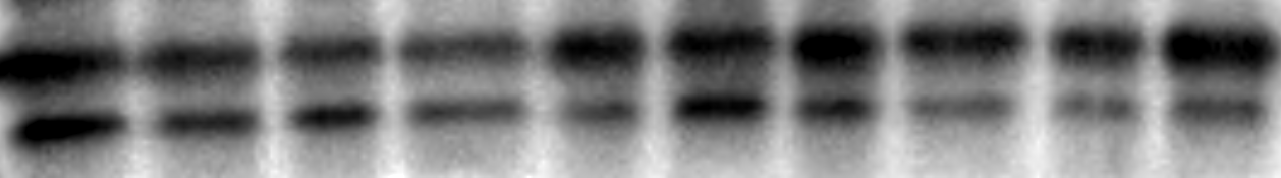


GAPDH band picture

**
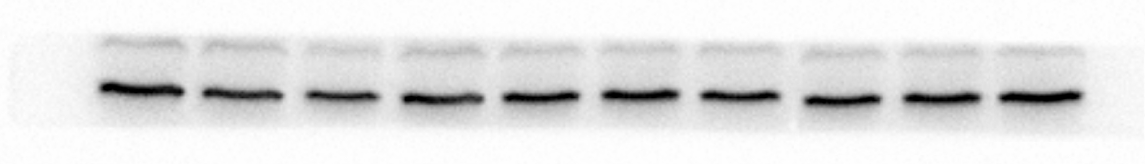
**
